# A Novel Hand-Held Spinning Platform with Centrifugal Microfluidics for Rapid, Cost-Effective Urinary Total Protein Detection at the Point of Care

**DOI:** 10.1021/acs.analchem.5c00930

**Published:** 2025-07-08

**Authors:** Wei Li, Jing Zhang, Junchen Liao, Mingze Zhu, Xinrui Wang, Xiang Zhou, Zhiqiang Ma, Mohamed Elhousseini Hilal, Song Lin Chua, Guangming Tan, Bryan P. Yan, Bee Luan Khoo

**Affiliations:** † Department of Biomedical Engineering, 53025 City University of Hong Kong, Hong Kong 999077, China; ‡ Hong Kong Center for Cerebro-Cardiovascular Health Engineering (COCHE), Hong Kong 999077, China; § College of Basic Medicine, Hebei University, Baoding, Hebei Province 071000, China; ∥ Department of Applied Biology and Chemical Technology, The Hong Kong Polytechnic University, Hong Kong 999077, China; ⊥ Division of Cardiology, Department of Medicine and Therapeutics, The Chinese University of Hong Kong, Prince of Wales Hospital, Hong Kong 999077, China; # City University of Hong Kong Shenzhen Research Institute (CityUSRI), Shenzhen 518057, China

## Abstract

Urinary total protein (UTP) serves as a critical biomarker for diagnosing and managing various diseases; however, current detection methods often face limitations regarding accessibility, cost, and operational complexity for point-of-care applications. This study presents a novel hand-held spinning platform with centrifugal microfluidics (HSP-C^M^), a groundbreaking technology designed for electricity-free, cost-effective UTP detection at a remarkably low cost of approximately 1.4 USD per test. The HSP-C^M^ integrates a 3D-printed spinning device that ensures uniform reagent distribution and a centrifugal microfluidic biochip capable of providing quantitative UTP analysis in a colorimetric, rapid (∼3 min), and user-friendly format. Notable innovations of the system include tunable hydrophilic–hydrophobic surface properties via microchannel aspect ratio adjustments, optimized optical conditions with an ultrafast exposure time of 1/100 s, and minimal sample volume requirements (5 μL), resulting in a detection limit as low as 3.49 μg/mL. The system demonstrated high throughput (n = 16) and strong correlation (0.9481) and agreement (90.32%) with gold-standard laboratory methods when validated using clinical urine samples from both healthy donors (n = 6) and myocardial infarction (MI) patients (n = 25). Furthermore, the HSP-C^M^ successfully identified clinically relevant UTP thresholds (>63.11 μg/mL) for distinguishing high-risk MI patients, achieving 100% sensitivity and specificity in patient classification. This novel platform presents a transformative solution for point-of-care proteinuria monitoring, offering significant potential for deployment in remote and resource-limited environments, where it could enhance disease detection, management, and patient outcomes through its noninvasive, rapid, and efficient capabilities.

## Graphical abstract

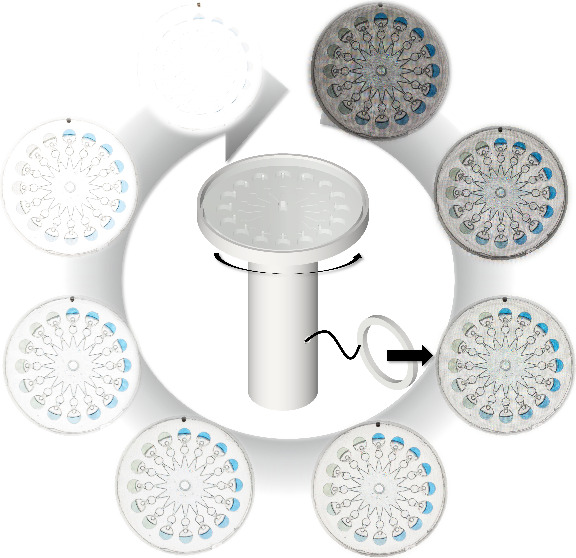

## Introduction

Urinary tests provide a noninvasive and cost-effective approach for screening, diagnosing, and monitoring diseases, offering distinct advantages over other biofluidic tests such as blood analyses.^1^ Proteinuria is an independent marker for assessing conditions such as chronic kidney disease,^2,3^ diabetes,^4^ and cardiovascular diseases^5,6^ and can be evaluated by measuring total albumin or protein levels.

Conventional methods for testing proteinuria include urine dipstick tests, urine protein-to-creatinine ratio (UPCR), urine albumin-to-creatinine ratio (UACR), and measurement of absolute urinary protein excretion from 24-h urine collection (Table S1). While urine dipstick tests are convenient and practical, they provide only qualitative or semiquantitative results. For more accurate patient stratification and personalized medicine, quantitative testing is essential. UPCR, commonly used to estimate daily protein excretion from spot urine samples, may be unreliable in patients with sarcopenia.^2^ Moreover, quantitative tests often require sophisticated and expensive instruments operated by trained professionals, which poses significant challenges in detecting and monitoring diseases in remote and resource-limited settings where access to medical facilities is limited but urgently needed. Thus, developing rapid, accurate, affordable, and portable disease diagnosis and monitoring techniques is paramount.

To address the limitations of conventional diagnostic methods, the World Health Organization has advocated for developing point-of-care (POC) technologies that adhere to the ASSURED criteria: Affordable, Sensitive, Specific, User-friendly, Rapid and robust, Equipment-free, and Deliverable. Various optical, magnetic, and colorimetric biosensors have recently been engineered to meet these stringent requirements.^7^ Among the promising approaches, microfluidics has gained prominence due to its minimal sample volume requirement and high experimental efficiency.^8–11^ Microfluidic techniques have advanced biomedical research, including establishing cellular models for biomedical investigation^12–15^ and POC biomarker detection.^16–19^ Traditional microfluidic devices depend on external pumps for reagent injection and removal; however, centrifugal microfluidics harnesses centrifugal forces, eliminating the need for such pumps.^20,21^ However, relying on electricity to power centrifugal microfluidic systems limits their applicability in resource-limited settings.

In this study, we presented the development of a hand-held spinning platform with centrifugal microfluidics (HSP-C^M^) designed to assess urinary protein levels quantitatively using colorimetric algorithmic analysis. The integrated system consisted of 3D-printed spinning devices, a disposable centrifugal microfluidic biochip, and optical devices, providing a comprehensive and streamlined solution. This system was characterized by its simplicity, cost-effectiveness (∼1.4 USD), rapid processing time (∼3 min), low sample volume requirement (5 μL), and high throughput capability (n = 16).

Validation of the HSP-C^M^ system was conducted using urine samples from healthy donors (n = 6) and patients after postmyocardial infarction (MI) surgery (n = 25). The results demonstrated a robust correlation (0.9481) and strong agreement (90.32%) with gold standard methods obtained from commercial plate readers, with high stability observed over ∼1 h. Additionally, we established clear thresholds (>63.11 μg/mL) for urinary protein levels in post-MI patients, enabling effective stratification of patients at high risk.

Our HSP-C^M^ system offers significant advantages over conventional methods, including user-friendliness, affordability, a low detection limit (3.49 μg/mL), and the capacity for high-throughput analysis (n = 16). The platform’s successful validation with disease patients, particularly those with MI, underscores its potential as a powerful tool for early screening and prognosis of various diseases. This system holds promise for assisting clinicians in making prompt and accurate decisions in clinical settings, particularly in resource-limited environments.

## Experimental Section

### Design and Working Principles of the HSP-C^M^ System

The HSP-C^M^ system comprised four essential components: a hand-held spinning device, a custom centrifugal microfluidic biochip for urinary protein analysis, an illumination apparatus, and a smartphone. The biochip comprised two layers for sample and solution loading and colorimetric reactions. Initially, clinical samples were pipetted into the sample well, which can simultaneously process up to 16 samples. The colorimetric reagent, Coomassie Brilliant Blue G-250 Dye, was introduced into the central chamber of the biochip, allowing for distribution into each sample well. Microholes (∼500 μm) on the reaction chamber facilitated air exchange within the microchannels and the sample loading function. Subsequently, the samples and reagent-loaded biochip were affixed to the hand-held spinning device. Upon pulling the hand ring, the rotating holder traversed the fixator, ensuring uniform distribution of the colorimetric reagent into the sample wells. The reagent then reacted with the tested samples for 3 min. Next, the biochip was placed on the illumination gadget, and the smartphone captured colorimetric results. Ultimately, quantitative measurements of urinary protein concentration were derived.

### Fabrication of the HSP-C^M^ System

The structure was designed via Autodesk Inventor and printed using VeroClear and SUP706 materials for rigidity and support. The dimensions of the spinning device are 52 mm × 52 mm × 78 mm (length × width × height), with a weight of ∼10 g. Similarly, the biochip molds were designed using Autodesk Inventor and printed using a 3D printer (Objet260 Connex3, Stratasys, USA) (Figure S1). The molds were baked for over 12 h to eliminate residual supporting materials. The biochip was fabricated using soft lithography techniques. Specifically, a mixture of PDMS (Sylgard 184 Silicone Elastomer Kit, Dow Corning, USA) in a 10:1 ratio (elastomer versus curing agent) was prepared and poured into the molds to create the desired patterns. The PDMS-filled molds were then subjected to vacuum treatment to remove any trapped air bubbles before being placed in an oven. Following baking at 70 °C for 2 h, the first PDMS layer, which featured reagent loading and venting holes, the solution wells, and the sample-reaction wells, was peeled off from the molds. The bottom glass surface was hydrophilic to facilitate smooth reagent flow through the microchannels. To assemble the biochip, the first PDMS and second glass layers underwent plasma treatment for 3 min at 700 mmtor. Finally, the assembled chip comprising the two layers was baked at 70 °C for 2 h to ensure proper binding.

### Calibration of the Optical Apparatus

For each assay, an iPad 2 (Apple, Inc., Cupertino, CA, USA) was set to its highest backlight intensity to provide consistent illumination. Adobe Lightroom, employing a professional shooting model, captured the colorimetric signals. The Bayer filter in smartphone camera sensors enables reliable optical imaging and accurate color sensing.^22,23^ The exposure time, International Standardization Organization (ISO), and white balance of the smartphone (iPhone 7, Apple, Inc., Cupertino, CA, USA) were critical parameters governing the amount of light reaching the image sensors, camera sensitivity, and color intensity adjustments, respectively. The white balance was set to automation to achieve equal balance across the red, green, and blue channel intensities. In contrast, the camera ISO was set to the minimal value (25) to minimize noise, and the exposure time ranged from 1/10 to 1/6400 s to optimize assay sensitivity. To overcome the influence of ambient light conditions and ensure consistent image quality with the appropriate field of view, a 3D-printed imaging setup (158.8 × 89.5 × 160 mm) was prepared (Figure S2).

### Colorimetric Assay for Total Protein Detection

The total protein quantification using a colorimetric assay was performed using a commercial kit (Bradford protein assay kit, PC0010, Solarbio) according to the manufacturer’s recommended protocols. The protein detection range was determined by the volume ratio of the tested samples, and the Coomassie Brilliant Blue G-250 Dye was diluted to the working concentration. The operating range was set from 0 to 2000 μg/mL, covering the range of clinical urinary protein levels (>300 μg/mL urine albumin). Briefly, upon incubation with the Coomassie dye for approximately 3 min, the color of the mixture changes from brown to blue, indicating the formation of a protein-dye complex. The smartphone captures an image of the biochip, and image processing algorithms are applied to extract the colorimetric information from the image. The intensity of the blue color was proportional to the concentration of total protein in the sample. Calibration curves were generated using known protein standards to establish a quantitative relationship between color intensity and protein concentration.

### Quantitative Detection of Urinary Protein by Image

In addition to direct visualization, urinary protein concentration was quantified through image analysis. The photos were captured using an iPhone 7 and processed using MATLAB software. With fixed biochip positioning and a predefined distance between the smartphone and biochip, color intensities from each sample well were extracted (10 × 10 pixels) based on the predetermined detection position. The pixel arrays representing the color channels (red, green, and blue) were separated, and the transmittance of the red channel was selected for subsequent analysis. To eliminate background noise, the red channel intensity of standards and samples was calibrated by subtracting the blank intensity, determining the calibrated absorbance of the red channel. The blank samples were tested and consisted of distilled water subjected to the same experimental procedures (i.e., the addition of Coomassie dye for the reaction). A calibration curve was generated by fitting the calibrated color intensity from the standards using a 4-PL fitting approach.^24^ Finally, the urinary protein concentration was calculated based on the fitting curve and the calibrated color intensity.

### Clinical Urine Sample Preparation

Urine samples from healthy donors (n = 6) were obtained from Innovative Research, while 25 urine samples from patients after MI were obtained. Ethical approval for the study was obtained from the Joint Chinese University of Hong Kong-New Territories East Cluster Clinical Research Ethics Committee (ref no. 2021.457). Urine samples from patients after MI surgery were collected and stored in plastic urine collection cups. The cups were transferred to the laboratory, aliquoted into 2 mL tubes, and frozen at −80 °C for future testing. Before testing, the urine samples were thawed to room temperature. The performance of the colorimetric-based detection method was evaluated by comparing the detected protein concentrations with those obtained using a commercial plate reader as the gold standard.

### Reference Urinary Protein Test (Spectrophotometer)

Calibrated standards and clinical samples (10 μL standards/sample and 200 μL colorimetric reagent) were added in triplicate to a 96-well transparent microtiter plate for a 3 min reaction. The plate was then read using a benchtop microplate reader (SpectraMax M5e Multi-Mode Microplate Reader, Molecular Devices) operating in absorbance mode with 594 nm light emission. Before testing, the plates were gently shaken for 5 s at room temperature.

### Statistical Analysis

MATLAB software was employed for statistical analysis. The absorbance and color intensity values of the standards and tested samples were adjusted by subtracting the signals from the blank samples (i.e., distilled water with Coomassie dye). A 4-PL fitting method was used to derive the best-fitting curve. Student’s *t* tests were employed to assess the associations between independent variables, and P values were calculated among different groups. ROC curves were constructed to evaluate the ability of the urinary concentration predictor to distinguish between healthy and patient samples. The cutoff value was determined using Youden’s index, which maximizes sensitivity and specificity. Sensitivity and specificity were calculated as the ratios of true positives to the sum of true positives plus false negatives and true negatives to the sum of true negatives plus false positives, respectively.

## Results and Discussion

### Development and Functionality of the Hand-Held Spinning Platform with a Centrifugal Microfluidics (HSP-C^M^) Platform for Diagnostic Testing in Resource-Limited Environments

Disease detection in resource-limited and remote areas is often hindered by transporting samples to centralized facilities, where advanced instruments and trained personnel are available (Figure 1a). This process can be time-consuming and may delay critical medical decisions. We developed an affordable hand-held spinning platform with centrifugal microfluidics (HSP-C^M^) to address these challenges and facilitate POC testing. This system delivers rapid and sensitive results and is user-friendly, eliminating the need for complex or electrical equipment.

**Figure 1:**
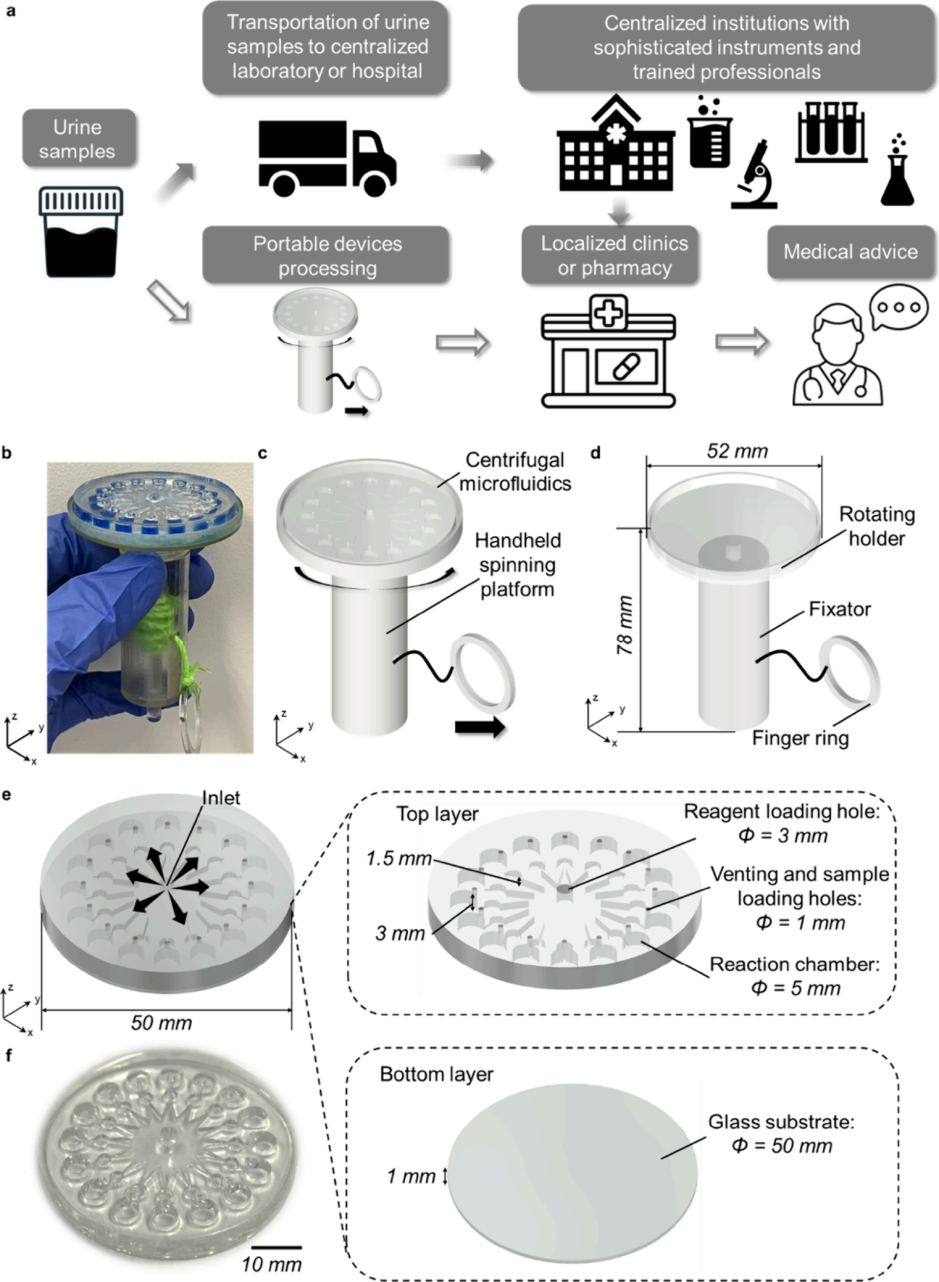
**The HSP-C** ^**M**^ **System for Point-of-Care Proteinuria Monitoring.** (a) In resource-limited or remote settings, sample detection is often delayed due to transporting samples to centralized laboratories or hospitals, where sophisticated instruments and trained professionals are required for analysis. This process is time-consuming, taking several days or weeks from sample collection to the final report, which is then sent back to local clinics or pharmacies for medical advice. In contrast, the HSP-C^M^ system enables rapid on-site proteinuria detection within approximately 3 min. (b) Photo of the HSP-C^M^ system, which includes a centrifugal microfluidic biochip loaded with samples and a hand-held 3D-printed spinning device for uniform reagent distribution. (c) Schematic diagram of the portable HSP-C^M^ system: the centrifugal microfluidic biochip is placed on a customized, manually operated hand-held spinning device. (d) 3D view of the custom-designed spinning device, with dimensions of 52 mm in diameter and 78 mm in height. When the user pulls the finger ring, the rotating holder spins the biochip, ensuring even distribution of the reagent across each reaction well. (e) The schematic diagram of the two-layer centrifugal microfluidic chip (50 mm diameter) was designed for efficient colorimetric analysis. (f) The photo of the centrifugal microfluidic biochip shows the integrated reaction wells for conducting colorimetric assays.

The HSP-C^M^ system consisted of custom-designed, manually operated spinning devices and a disposable centrifugal microfluidic chip optimized for automated colorimetric assays (Figures 1 **b and** [Fig fig1] **c**). The hand-held spinning device was compact, with dimensions of 52 mm in diameter and 78 mm in height (Figure 1d). During operation, the centrifugal microfluidic chip was placed on the 3D-printed spinning device, which, when spun, evenly distributes the reagent across the chip into each reaction well, facilitating the colorimetric assay. This portable, easy-to-use system was particularly suited for deployment in settings with limited access to conventional laboratory infrastructure.

The two-layer centrifugal microfluidic chip, with a diameter of 50 mm and a height of approximately 4 mm, was designed to facilitate efficient colorimetric absorption detection (Figures 1 **e-** [Fig fig1] **f**). The top layer of the chip included a reagent loading port for introducing the colorimetric dye, along with 16 reaction chambers for conducting the colorimetric reactions. Each reaction chamber had a venting and sample loading hole to maintain air balance during the spinning process and while loading the test samples. These venting holes, each with a diameter of 500 μm, also functioned as sample-loading ports.

The reagent chamber was designed to hold approximately 400 μL of the solution, while the 16 reaction wells were configured to contain the assay solution and produce colorimetric readouts. The microchannels in the top layer had dimensions of 500 μm in width and height. These microchannel patterns were fabricated using customized 3D-printed molds, with subsequent polydimethylsiloxane (PDMS) demolding and solidification (Figure S1). This design ensured precise fluid handling and reliable colorimetric analysis within the microfluidic chip.

The bottom layer of the chip was composed of a flat glass substrate, which provided a hydrophilic surface to ensure uniform solution distribution. In contrast, the top layer comprised hydrophobic PDMS to prevent fluid from floating within the microchannels. By carefully adjusting the aspect ratio of the microchannels, the fluid flow was precisely regulated or halted as needed. The detailed design and fabrication procedures for the biochip were outlined in the Methods section.

### Operational Validation and Uniformity Analysis of Reagent Distribution in the HSP-C^M^ Platform

The HSP-C^M^ system was developed as a POC device for detecting and monitoring proteinuria, particularly tailored for application in remote and resource-limited settings. This system offered a cost-effective solution with rapid result delivery, user-friendly operation, and independence from external power sources. The estimated chip and assay cost for analyzing 16 samples using the HSP-C^M^ system was approximately USD 1.4 (Tables S2 and S3), with the potential for further cost reduction through scaled-up production.

To verify the spatial solution distribution for the HSP-C^M^ system, we delineated the primary steps involved: (i) reagent loading, (ii) device spinning, and (iii) results readout (Figure 2a). Initially, the colorimetric reagent was introduced through the reagent-loading wells, followed by positioning on the spinning device. After manually spinning briefly, the reagent was dispensed into 16 reaction chambers within seconds and reacted with the loaded samples in each chamber. After waiting for ∼3 min, the color intensities in each reaction well were assessed quantitatively using a smartphone.

**Figure 2:**
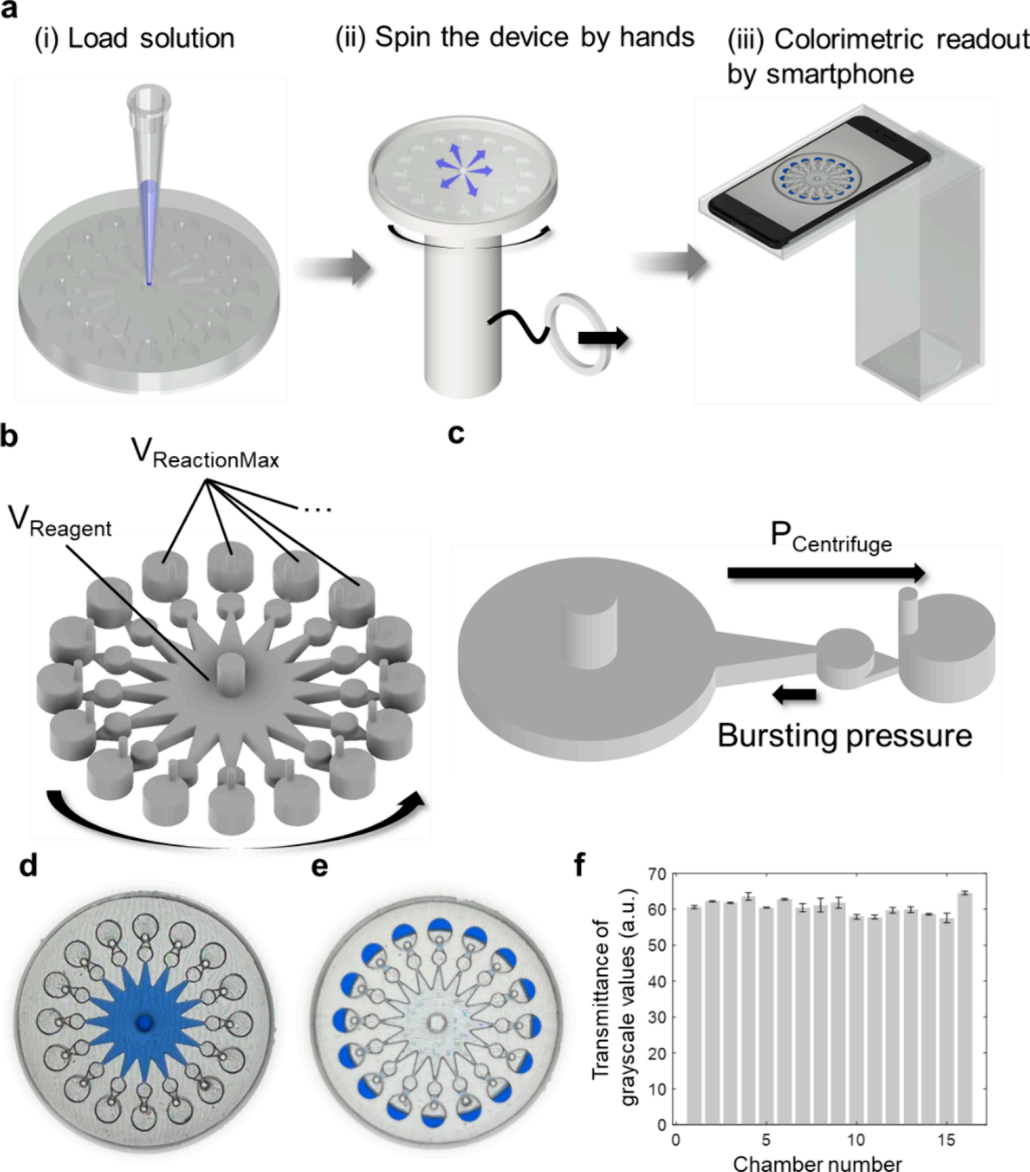
**Characterization of the HSP-C** ^**M**^ **System Operation and Reagent Distribution by Colorimetric Analysis.** (a) Flowchart of the user procedure for the HSP-C^M^ system. Colorimetric dyes were injected into the respective chambers, and the centrifugal microfluidic biochip was placed on the 3D-printed spinning device. After manual spinning, the reagent was distributed into each reaction chamber to react with the loaded samples for ∼ 3 min, followed by result capture using a smartphone. (b) Schematic diagram of the microchannel structure of the centrifugal biochip, comprising 16 reaction units designed for sample loading, reagent dispensing, and colorimetric assays. (c) Schematic of one microchannel unit showing the effects of centrifugal forces during spinning, which overcome the bursting pressure to facilitate reagent distribution. (d) and (e) Photographs of the centrifugal biochip: (d) after reagent loading and (e) after reagent distribution, demonstrating uniform spreading of the solution across the reaction wells. (f) Uniformity analysis of the solution distribution in the 16 reaction wells was assessed by measuring the transmittance of grayscale values, indicating a consistent reagent distribution with a low coefficient of variation (CV = 3.30%).

The UTP chip comprised 16 units for sample loading, reagent dispensing, and reaction (Figure 2b). After loading the reagent into the chamber, the volume was calculated using the following equation:

$$V_{Reagent}=nh_{1}{R_{1}}^{2}\;\sin\left(\frac{180^{{}^{\circ}}}{n}\right)\left(1+\frac{\sin\frac{180^{{}^{\circ}}}{n}}{\tan\alpha_{1}}\right)$$ 1

The volume of the reaction chamber, denoted as *V*_*ReactionMax*_ can be determined by the equation:

$$V_{Reaction\mathrm{Max}}=nh_{2}{R_{4}}^{2}\left[\pi -\frac{2\pi \;\cos^{-1}\frac{R_{4}-2R_{3}}{R_{4}}}{360^{{}^{\circ}}}+\frac{1}{2}\;\sin\left(2\;\cos^{-1}\frac{R_{4}-2R_{3}}{R_{4}}\right)\right]$$ 2

In the above eqs 1 **and (** [Disp-formula eq2] **)**, *n* represents the number of distribution channels (*n* = 16), *h* signifies the height of the channel (*h*_*1*_ = 1.5 mm, *h*_*2*_ = 3 mm), *R*_*1*_ corresponds to a portion of the sample chamber radius, and *R*_*3*_ and *R*_*4*_ represent the vent hole and reaction chamber radius, respectively (*R*_*3*_ = 0.5 mm, *R*_*4*_ = 2.5 mm). *α*_*1*_ corresponds to the tangential angles of the side walls (*α*_*1*_ = 11.55°) (Figure S3a). The total volume of the 16 reaction chambers (*V*_*ReactionMax*_ = 808 μL) exceeds the reagent loading volume (*V*_*Reagent*_ = 412 μL) to ensure no reaction solution splashes from the vent holes. from the vent holes.

The process entailed two main stages. The tested samples and reagent solution were loaded into their respective chambers in the first stage. The reagent remained in place due to the bursting pressure valves. Specifically, the bursting pressure of the capillary bursting valves was calculated as

$$BP=-\gamma \left\{\frac{\cos\varphi_{AGlass}+\cos\varphi_{APDMS}}{h_{1}}+\frac{2\;\cos\left[\min\left(\varphi_{APDMS}+\alpha_{1}+\alpha_{2}\right),180^{{}^{\circ}}\right]}{W_{2}}\right\}$$ 3

where γ represents the surface tension of the solution (γ = 0.072 N/m for water), φ_*AGlas*s_ is the advancing contact angle of the solution on the glass (φ_*AGlas*s_ = 16.8 ± 3.2° for water), *φ*_*APDMS*_ is the advancing contact angle of the solution on the PDMS (*φ*_*APDMS*_ = 125 ± 2° for water), *W*_*2*_ and *h*_*1*_ denotes the width and height of the diverging section (*W*_*2*_ = 0.5 mm, *h*_*1*_ = 1.5 mm), and *α*_*1*_ and *α*_*2*_ correspond to the tangential angles of the side walls (*α*_*1*_ = 11.55°, *α*_*2*_ ≈ 90°) (eq 3).^25–28^

To disrupt the balance and facilitate the movement of the reagent into the reaction chamber for colorimetric assays, centrifugal forces were introduced using our homemade hand-held spinning platform (Figure 2c). The centrifugal force was calculated as

$$P_{Centrifuge}=\frac{1}{2}\rho \omega^{2}{R_{2}}^{2}$$ 4

Where ω represents the angular velocity of the hand-held spinning platform, and *R*_*2*_ denotes the distance between the liquid and the center of the spinning platform (*R*_*2*_ = 11.78 mm) (Figure S3a). During spinning, the centrifugal pressure disturbed the bursting pressure, allowing the solution to move alongside the tested samples into the reaction chamber.

To prevent solution splashing outside the sample loading holes caused by the hydrophobic interface, the microchannels were intentionally designed with a sudden reduction in aspect ratio, transitioning from a higher to a lower aspect ratio to enhance hydrophilicity (Figure S3b). This design adjustment effectively redirected the dominant capillary forces from the lateral surfaces to the top and bottom surfaces. The hydrophilic nature of the glass substrate proved more influential than the hydrophobic properties of the PDMS top layer. As a result, capillary forces from the PDMS chamber walls facilitated the mixing and retention of reagents and samples within the reaction chamber.

To assess the spatial distribution of reagents within the HSP-C^M^ system, a color dye (blue ink) was introduced through the inlet (Figure 2d), and the solution was allowed to remain in the microchannels. The devices were then spun to facilitate the distribution of the solution (Figure 2e). Analysis revealed a relatively homogeneous color distribution across the 16 reaction wells. This uniformity was further confirmed by quantifying the intensities of grayscale values (Figure 2f). The low and consistent standard deviations observed in the grayscale value (60.67 ± 2.00) and coefficient of variation (CV) (3.30%) indicated a uniform solution distribution.

### Calibration of Smartphone Camera Parameters for Quantitative Analysis in the HSP-C^M^ System

To optimize the smartphone camera’s exposure time (ET) and color channels for sensitive detection, an analysis of the color intensity emitted by the illumination apparatus was conducted (Figure 3a). The apparatus was set to maximum illumination intensity to minimize interference during ET optimization. A range of 10 ETs (1/6400, 1/3200, 1/1600, 1/800, 1/400, 1/200, 1/100, 1/50, 1/10, and 1/5 s) was tested on the smartphone camera. The consistency of intensity levels across the red, green, and blue channels and the very low standard deviations for each channel indicated uniform and stable illumination. Saturation of color intensity, where color intensity reached 255, was observed at ETs of 1/5 and 1/10 s, resulting in images that appeared pure white. Conversely, as the ET decreased, the color intensities diminished due to reduced light capture by the smartphone’s image sensor.

**Figure 3:**
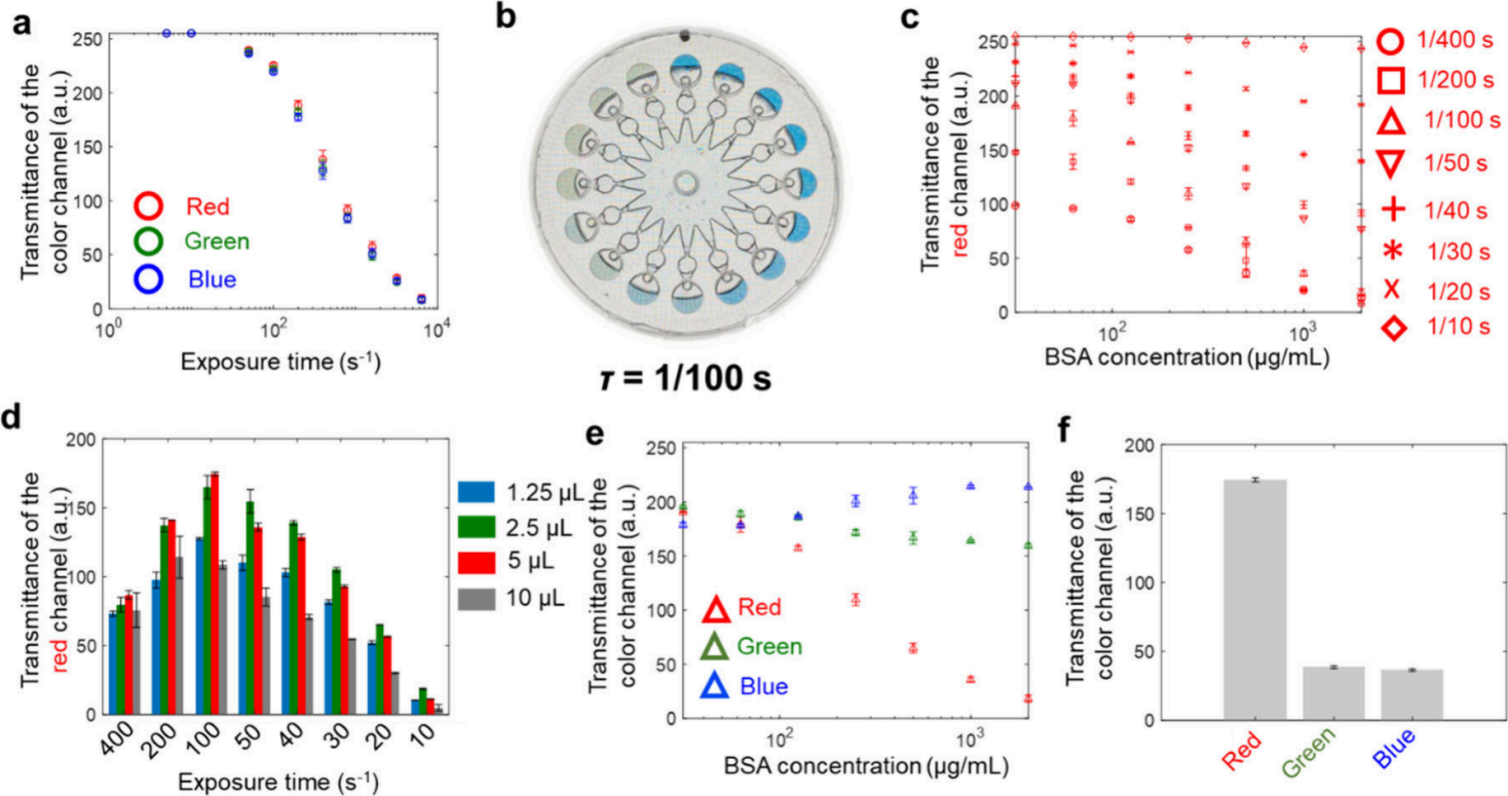
**Optimization of the HSP-C** ^**M**^ **System for Illumination and Smartphone Image Capture.** (a) The transmittance intensity of the illumination from the device showed a decrease in color intensity as the smartphone exposure time (ET) was reduced. (b) Representative images of standard BSA samples captured by the smartphone with the optimal exposure time of 1/100 s and a sample volume of 5 μL. (c) There is an inverse correlation between the transmittance of the red channel and BSA concentration at different exposure times, indicating higher BSA concentrations result in lower transmittance. (d) Dynamic range of transmittance in the red channel across different sample volumes (1.25 μL, 2.5 μL, 5 μL, and 10 μL) at various exposure times (τ = 1/400, 1/200, 1/100, 1/50, 1/40, 1/30, 1/20, and 1/10 s). (e) The transmittance of the red, green, and blue channels with BSA concentration showed a decrease in red channel transmittance with increased BSA. In contrast, green and blue channels exhibited minimal intensity changes. (f) Comparison of the dynamic ranges for the red, green, and blue channels, with the red channel demonstrating the broadest dynamic range (174.33 ± 1.49), significantly higher than those of the green (38.48 ± 0.91) and blue (36.38 ± 0.94) channels.

To determine optimal parameters for the HSP-C^M^ system, eight standard BSA samples with concentrations of 2000, 1000, 500, 250, 125, 62.5, 31.25, and 0 μg/mL (blank) were prepared with G250 reagent and loaded into the biochip (Figure S4). Images were captured using ETs of 1/400, 1/200, 1/100, 1/50, 1/40, 1/30, 1/20, and 1/10 s to identify the optimal ET (Figures 3 **b and** S5). Additionally, standard samples with volumes of 1.25 μL, 2.5 μL, 5 μL, and 10 μL were analyzed to determine the optimal sample loading volume (Figures 3 **b and** S6).

The color intensity in the reaction wells was quantified by extracting and calculating the transmittance of the red channel (Figures 3 **c and** S7). An inverse correlation was observed between BSA concentration and red channel transmittance, where higher BSA concentrations led to lower transmittance and higher optical absorbance. The dynamic transmittance range varied with different ETs (Figure 3d). Among the tested ETs, 1/100 s yielded the highest dynamic range (143.82 ± 26.87) across different sample volumes, making it the optimal exposure time for sensitive detection. Furthermore, a sample volume of 5 μL provided the highest dynamic transmittance range (174.33 ± 1.49) compared with both lower and higher volumes, establishing it as the optimal loading volume for further analysis.

Similarly, transmittance values were measured for the green and blue channels and compared with those from the red channel (Figure 3e). However, the green and blue channels exhibited considerable fluctuations and limited dynamic ranges. Among the three color channels, the red channel demonstrated the broadest dynamic range (174.33 ± 1.49) and the strongest correlation with analyte concentration (Figure 3f). Consequently, the red channel was selected for quantitative analysis of UTP, in alignment with the Beer–Lambert Law and color complementarity principles. In conclusion, the red channel, an optimized exposure time of 1/100 s, and a sample volume of 5 μL were the optimal parameters for quantitative analysis using the smartphone camera.

### Calibration and Sensitivity Characterization of the HSP-C^M^ System for Protein Detection

To calibrate the HSP-C^M^ system, eight standard BSA samples (2000, 1000, 500, 250, 125, 62.5, 31.25 μg/mL, and a blank) were analyzed using red channel transmittance measurements (Figure 4a). An increase in BSA concentration resulted in a corresponding decrease in red channel transmittance, attributed to BSA’s higher light absorbance capacity at elevated concentrations. To mitigate background signal interference, the absorbance intensities of the standards were calibrated (Figure S8). The 4-parameter logistic (4-PL) model was used to fit the calibration curve, following established protocols for quantitative analysis.^24^ The equation described the relationship between standard BSA concentration and red channel absorbance intensity after correcting for the blank sample’s color intensity (Figure 4b)

$$y=\frac{B-A}{1+{\left(\frac{x}{C}\right)}^{D}}+A$$ 5

**Figure 4:**
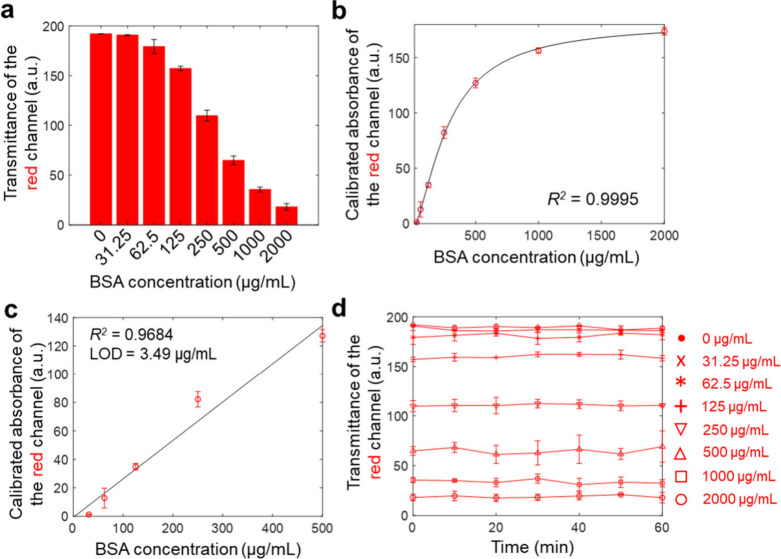
**Calibration of the HSP-C** ^**M**^ **System Using BSA Standards.** (a) Red channel transmittance measurements for standard BSA samples at various concentrations. (b) Calibration curve of the red channel absorbance versus standard BSA concentration. The correlation coefficient (R^2^) is shown, indicating the fit of the 4-parameter logistic model. (c) Linear dynamic range of the calibration curve for BSA detection, with the correlation coefficient (R^2^) and detection limit indicated on the plot. (d) Stability analysis of standard protein samples shows that the red channel transmittance remained stable over time (CV < 6%), confirming the consistency and reliability of the system for prolonged use.

Here, *y* represents the responses, *x* denotes the BSA concentration, *A* and *B* correspond to the maximum and minimum absorbance values, and *C* and *D* signify the inflection point and the slope factor of the curve at the inflection point, respectively. The determined values for *A*, *B*, *C*, and *D* were −5.24, 181.27, 278.59, and −1.53, respectively (eq 5). The correlation coefficient (R^2^) obtained was 0.9995, indicating a significant fit of the proposed model to the standard BSA concentration and suitability for further quantitative analysis of actual clinical urine samples. The calibration curve demonstrated a detection range from 31.25 to 2000 μg/mL, encompassing the clinical range of urinary total protein levels (i.e., <300 μg/mL).^29^

The HSP-C^M^ system exhibited a linear regression formula: $y\left(0-500\frac{\mathrm{\mu g}}{\mathrm{mL}}\right)=-0.76+0.27x$ has a detection limit of 3.49 μg/mL, a limit of quantification of 5.05 μg/mL, and linear dynamic ranges of 5.05–500 μg/mL (Figure 4c). This detection limit was approximately 6.45 times lower than that of commercial plate readers (22.53 μg/mL), and the HSP-C^M^ required half the sample volume, reflecting a lower workload and potential for use in benchtop, instrument-free settings (Figure S9). The achieved low detection limit confirmed the successful optimization of the HSP-C^M^ system parameters.

We examined the reaction time across various standard BSA concentrations. The transmittance of the red channel was measured for seven standard BSA samples at 10 min intervals (Figure 4d). The color intensities remained stable for up to 1 h, with minimal fluctuation observed in the high-concentration samples, including 2000 μg/mL (18.94 ± 1.14, CV = 6%), 1000 μg/mL (34.00 ± 1.96, CV = 5.76%), and 500 μg/mL (64.97 ± 2.53, CV = 3.90%) samples throughout the experiment. These results underscore the high stability of the HSP-C^M^ system in detecting UTP levels. Notably, the reaction product was effectively retained within the microchannel due to capillary pressure induced by the PDMS layer, which prevented backflow and cross-contamination between samples.

### Clinical Validation of the HSP-C^M^ System for Urinary Total Protein (UTP) Detection

Subsequently, the HSP-C^M^ system detected UTP levels in clinical samples. A total of 31 urine samples were collected, including those from healthy donors (n = 6) and patients following MI surgery (n = 25), based on their clinical diagnoses from the hospital. All urine samples of MI patients were collected post-surgery.

The samples were analyzed using the HSP-C^M^ system and a commercial plate reader in a 96-well plate format. Following the standard testing protocol, 5 μL of urine and 412 μL of G250 reagent were loaded into the biochip (Figure 5a). The finger ring was then pulled to distribute the reagents into various reaction chambers for colorimetric analysis, and the reaction was allowed to proceed for 3 min. The reaction product was subsequently captured using a smartphone with an exposure time of 1/100 s. The UTP concentration in the clinical samples was determined based on the calibrated absorbance of the red channel and the established calibration curve. The HSP-C^M^ system simultaneously tested 16 urine samples (Figure S10).

**Figure 5:**
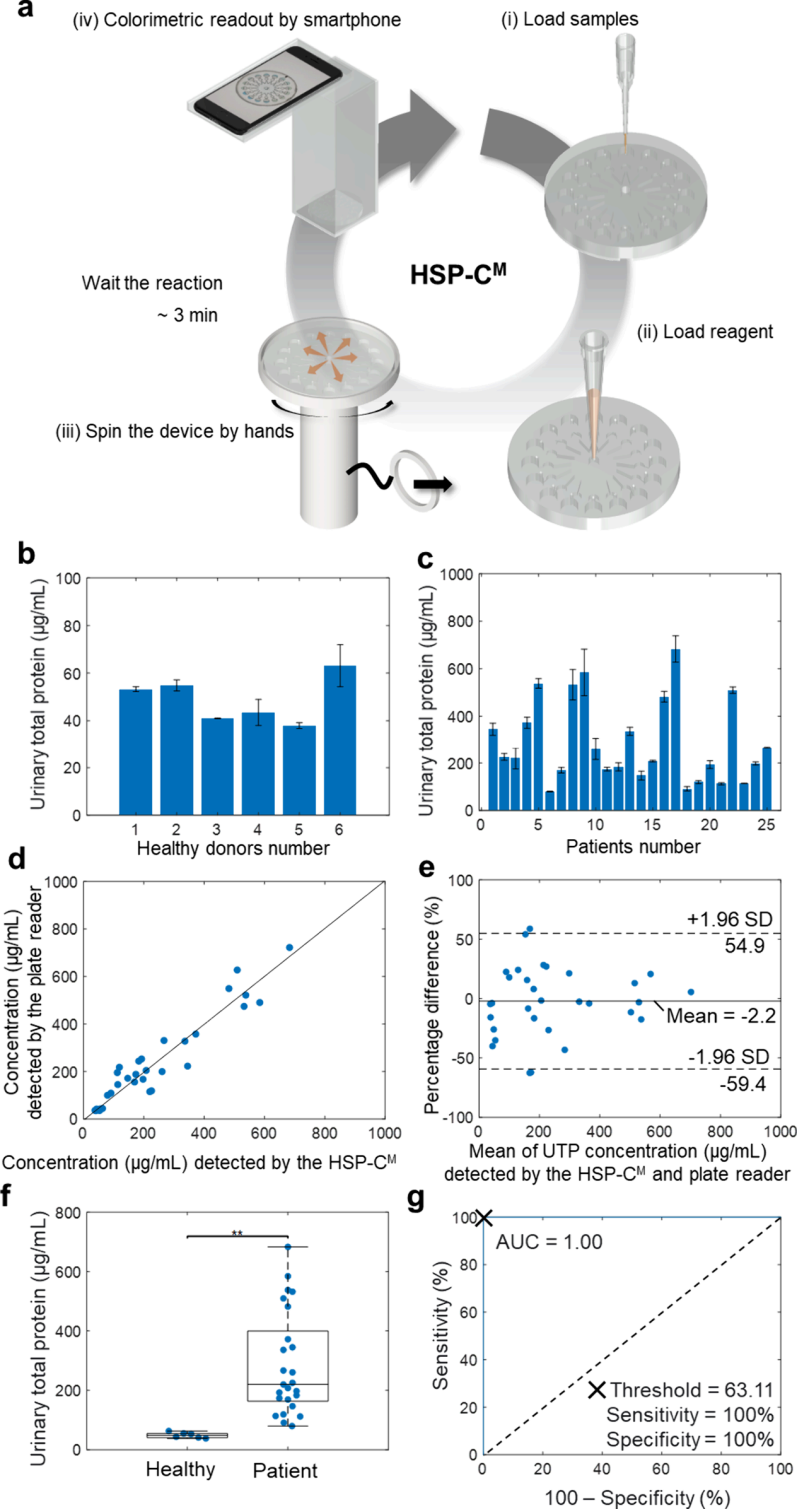
**Clinical Testing and Validation of the HSP-C** ^**M**^ **System for UTP Detection.** (a) Flowchart outlining the procedure for clinical sample analysis using the HSP-C^M^ system, including sample and reagent loading, device spinning for reagent distribution, and colorimetric readout captured via smartphone with the optimized exposure time (1/100 s). (b) and (c) The measured urinary total protein (UTP) levels in samples from healthy donors (*n* = 6) and postmyocardial infarction (MI) patients (*n* = 25). (d) Correlation analysis between the UTP concentrations obtained from the HSP-C^M^ system and the commercial plate reader for both healthy donors (*n* = 6) and MI patients (*n* = 25), with the regression line shown (solid black). (e) Bland-Altman analysis comparing UTP results from the HSP-C^M^ system and the commercial plate reader. The solid line indicates the mean difference, while the dotted lines represent the 95% limits of agreement. (f) Boxplot comparing UTP concentrations between healthy donors (48.94 ± 8.83 μg/mL) and MI patients (285.61 ± 170.78 μg/mL). Statistical significance is indicated by ** (*p* ≤ 0.01). (g) Receiver Operating Characteristic (ROC) analysis distinguishes healthy and MI patient samples based on UTP concentration, showing sensitivity, specificity, and area under the curve (AUC) values.

UTP levels in the urine samples from healthy donors were significantly lower than those from MI patients (Figures 5 **b and** [Fig fig5] **c**). To compare the results obtained with the HSP-C^M^ system to those from the commercial plate reader (Figure S11), we assessed the correlation between the quantification results of healthy donors and patients after MI surgery from both methods (Figure 5d). The linear regression model yielded the formula

y=−5.31+1.01x 6

with a Spearman correlation coefficient of 0.902, denoting a substantial monotonic correlation between outcomes from the two techniques based on Passing-Bablok regression analysis.^30^ Furthermore, Lin’s concordance correlation coefficient was computed at 0.9481, signifying a robust correlation between results obtained from the HSP-C^M^ system and the commercial plate reader.^31^

Additionally, a Bland-Altman analysis was conducted to evaluate the agreement and assess bias errors between the results from the HSP-C^M^ system and the gold standard, the commercial plate reader (Figure 5e).^32^ This analysis expected most differences to fall within the range of δ ± 1.96s, where δ is the mean difference and s is the standard deviation of the differences. The results showed that 90.32% of the data points for healthy donors (n = 6) and post-MI patients (n = 25) fell within the limits of agreement. The mean bias was −2.2%, with 95% confidence intervals ranging from −59.4% to 54.9%, indicating that discrepancies between the two methods tended to increase with higher UTP levels in clinical samples.

Lin’s concordance correlation coefficient and Bland-Altman analyses collectively highlighted a robust correlation of 0.9481 and a 90.32% agreement between the HSP-C^M^ system and the commercial plate reader. These findings suggested that the HSP-C^M^ system is a valuable tool for quantitative point-of-care monitoring of proteinuria.

To explore the potential clinical relevance of UTP levels in differentiating between healthy donors (n = 6) and patients following MI (n = 25), we compared UTP concentrations across the two cohorts and established distinct thresholds using receiver operating characteristic (ROC) analysis. Our findings demonstrated that UTP levels were significantly elevated (*p* < 0.01) in the patient group relative to the healthy donors (Figure 5f), suggesting that elevated UTP levels are associated with an increased likelihood of unhealthy.

Distinct thresholds for classifying the two cohorts were determined using ROC curves (Figure 5g). The area under the curve (AUC) values were calculated as 1, indicating the optimal performance of UTP in differentiating healthy donors from MI patients. Thresholds were established based on Youden’s index, which maximizes the sum of sensitivity and specificity. At a 63.11 μg/mL threshold, UTP demonstrated perfect sensitivity (100%) and specificity (100%) for classifying MI patients. These high AUC values, combined with ideal sensitivity and specificity, affirm the clinical significance of UTP in distinguishing healthy individuals from those at high risk of MI, using a well-defined threshold (>63.11 μg/mL) for identifying patients at elevated risk. Comparable results were obtained using the commercial plate reader (Figure S12). These findings underscore the utility of the HSP-C^M^ system as an effective tool for point-of-care disease monitoring.

## Conclusions

We developed a portable, electricity-free hand-held urinalysis platform for quantifying UTP levels in clinical patients. This system utilized a disposable centrifugal microfluidic chip and a custom-designed hand-held spinning device, ensuring uniform sample distribution by adjusting the microchannels’ aspect ratio. The calibration curve established with standard proteins demonstrates exceptional stability and a low detection limit under optimal system parameters, including exposure time, sample volume, and color channels. The quantification results from our method showed a significant correlation and agreement with those obtained using a commercially available plate reader. Furthermore, our study indicated significantly elevated UTP levels in the urine of patients with MI compared to healthy controls, with clear threshold values established for distinguishing between these cohorts. This portable urinalysis platform has considerable potential for point-of-care disease monitoring.

## Supplementary Material



## Notes

Additional discussion and a glossary of acronyms; figures of centrifugal microfluidics mold and smartphone-based imaging setup (S1, S2), chip design and loading process illustration (S3, S4), optimization results of exposure time and sample volume (S5, S6), transmittance profile with protein concentration (S7), schematic of algorithmic analysis workflow (S8), calibration curves and colorimetric performance (S9), representative colorimetric images and correlation plots (S10–S12), smartphone performance comparison and imaging reproducibility (S13–S16); tables summarizing comparisons of conventional urinary protein detection methods (S1), microfluidic chip cost breakdown (S2), per-assay cost analysis (S3), benchmarking against other centrifugal microfluidic platforms (S4), and comparison with existing portable urinary protein/albumin detection technologies (S5) (PDF)
