# Supplementary material for: A Novel Hand-Held Spinning Platform with Centrifugal Microfluidics for Rapid, Cost-Effective Urinary Total Protein Detection at the Point of Care
Source: Anal Chem. 2025 Jul 8;97(28):15049–59. doi: 10.1021/acs.analchem.5c00930 (PMC12291037; doi:10.1021/acs.analchem.5c00930)
Supplement: Supplementary file 1 [file ac5c00930_si_001.pdf]

Supporting Information

A Novel Handheld Spinning Platform with Centrifugal Microfluidics for Rapid, Cost-Effective Urinary Total Protein Detection at the Point of Care

Wei Li [1, 2], Jing Zhang [3], Junchen Liao [1], Mingze Zhu [1, 2], Xinrui Wang [1, 2], Xiang Zhou [1], Zhiqiang Ma [1, 2], Mohamed Elhousseini Hilal [1, 2], Song Lin Chua [4], Guangming Tan [5], Bryan P. Yan [2, 5], Bee Luan Khoo [1, 2, 6] *

[1] Department of Biomedical Engineering, City University of Hong Kong, Hong Kong, 999077, China

[2] Hong Kong Center for Cerebro-Cardiovascular Health Engineering (COCHE), Hong Kong, 999077, China

[3] College of Basic Medicine, Hebei University, Baoding, Hebei Province, 071000, China

[4] Department of Applied Biology and Chemical Technology, The Hong Kong Polytechnic University, Hong Kong, 999077, China

[5] Division of Cardiology, Department of Medicine and Therapeutics, The Chinese University of Hong Kong, Prince of Wales Hospital, Hong Kong, 999077, China

[6] City University of Hong Kong Shenzhen Research Institute (CityUSRI), Shenzhen, 518057, China

Table of Contents

List of Supplementary Figures S-3
List of Supplementary Tables........ S-4
Additional Discussions........ S-5
Acronyms S-8
Supplementary Figures........ S-9
Supplementary Tables S-25
References S-30

List of Supplementary Figures

Figure S1. Centrifugal microfluidics mold.

Figure S2. Design of the smartphone-based imaging setup.

Figure S3. Illustration of centrifugal microfluidics from (a) top view and (b) side view.

Figure S4. (a) Sample and (b) reagent loading into the HSP-C^M to test samples.

Figure S5. Representative photos of the centrifugal biochip of standards imaged under different exposure times (i.e., 1/400, 1/200, 1/50, 1/40, 1/30, 1/20, and 1/10 s).

Figure S6. Representative photos of the centrifugal biochip of standards with a sample volume of (a) 1.25 µL, (b) 2.5 µL, and (c) 10 µL, imaged under the exposure time of 1/100 s.

Figure S7. The transmittance of the red channel with the BSA concentration increased.

Figure S8. Flowchart of the colorimetric algorithmic analysis procedures.

Figure S9. The calibration curve of standards was detected by the plate reader with a detection limit of 22.53 µg/mL and linear dynamic ranges of 75.90 – 500 µg/mL.

Figure S10. Representative photos of the colorimetric results detected from (a) the healthy cohort and (b) the patient cohort.

Figure S11. Clinical testing results of the UTP levels from (a) healthy donors (n = 6) and (b) patients after myocardial infarction (MI) surgery (n = 25) detected by commercial plate reader.

Figure S12. Correlation analysis of clinical urine samples detected by the commercial plate reader.

Figure S13. The transmittance intensity of the illumination detected by (a) iPhone 13 Pro and (b) Xiaomi Mi 10T Pro.

Figure S14. Established calibration curves of standard samples from (a) iPhone 13 Pro and (b) Xiaomi Mi 10T Pro with exposure times of 1/100 s.

Figure S15. Representative photographs of solution distribution tested by three different operators (a)–(c).

Figure S16. Detected transmittance results were based on grayscale values (CV = 2.64 ± 0.65%) from three operators (n = 3).

List of Supplementary Tables

Table S1. Comparison of advantages and limitations of conventional techniques for proteinuria measurement.

Table S2. The disposable centrifugal microfluidic chip cost.

Table S3. Assay cost per chip.

Table S4. Comparison of existing centrifugal microfluidic techniques with our system.

Table S5. Comparison of existing techniques for portable and quantitative urinary protein/albumin test.

Additional Discussions

To power the centrifugal forces without electricity, an innovative centrifugal microfluidic device inspired by the ancient whirligig led to the creation of a hand-powered paper centrifuge capable of achieving ultrafast speeds (~125,000 r.p.m.) at an ultra-low cost of approximately 20 cents [1]. Building on this concept, several toy-inspired devices have been developed to facilitate centrifugal microfluidic spinning. For example, a hand-powered centrifugal microfluidic system inspired by the spinning top was devised for nucleic acid detection using loop-mediated isothermal amplification [2] and for serum separation in COVID-19 immunoassays [3]. Another adaptation used a commercial hand fan to generate centrifugal force, which enabled antibiotic susceptibility testing when coupled with microfluidics [4] and antibody quantification [5]. Additionally, the customization of a fidget spinner with centrifugal microfluidics and fluid-assisted separation technology (FAST) allowed for rapid (~50 minutes), uniform, and efficient (>100-fold concentration) point-of-care diagnosis of urinary tract infections [6]. Centrifugal microfluidics assisted by FAST has proven valuable not only in extreme point-of-care testing but also in personalized medicine [7,8], supporting the detection of various tumor-related biomarkers, including circulating tumor cells [9], circulating tumor DNA [10], and extracellular vesicles [11].

Besides, traditional centrifugal microfluidics have predominantly been fabricated using polymethylmethacrylate (PMMA). The inherent hydrophobicity and fabrication limitations of PMMA, particularly achieving high aspect ratio microchannels, have necessitated using multiple layers and complex valve structures during centrifugal chip formation. The manual assembly of these layers is often labor-intensive and time-consuming, increasing the risk of errors during fabrication [12]. Recent advancements in 3D printing technologies, such as stereolithography, multi-jet modeling, and fused deposition modeling, have significantly streamlined the challenging processes associated with traditional lithography, opening new avenues for microfluidic mold fabrication [13-15].

In this study, we harnessed the potential of 3D printing to successfully fabricate a single PDMS-based microfluidic layer with an intricate 3D structure designed for fluidic delivery. By optimizing the aspect ratio of the microchannels within the PDMS and glass substrates, we achieved relatively tunable hydrophilic and hydrophobic surfaces, which facilitated fluid trapping and ensured uniform reagent distribution. This innovative approach enhances the efficiency of microfluidic chip production and improves the precision and reliability of

centrifugal microfluidics, positioning it as a more viable option for various biomedical applications.

Consequently, when juxtaposed with alternative centrifugal microfluidics (**Table S4**), our platform exhibited three distinct advantages as an exceptional screening instrument: (i) the absence of electrical dependency in the handheld spinning platform, rendering it well-suited for utilization in resource-constrained environments; (ii) the capacity for simultaneous processing of 16 samples, ensuring high throughput; and (iii) streamlined fabrication owing to a single fluidic layer housing intricate structures, thereby rendering the HSP-C^M ideal for POC testing applications.

Proteinuria is a critical biomarker for monitoring urinary tract and kidney diseases, cardiovascular diseases, and various cancers. Elevated levels of proteinuria in patients are linked with an increased risk of cardiovascular conditions due to generalized vascular endothelial dysfunction [16,17], inflammation [18], thrombogenic factors [19], and insulin resistance [20]. In oncological contexts, proteinuria may arise from using anti-angiogenic agents in cancer patients [21]. These associations underscore the significance of urinary total protein levels in diagnosing and managing cardiovascular and cancer-related diseases.

While urinary albumin is a crucial marker for proteinuria, relying solely on albumin testing may fail to detect tubular proteinuria in specific cases [22]. Urinary total protein is an effective indicator for monitoring proteinuria, particularly in diabetic patients [23,24]. Currently, clinical urinalysis typically involves 24-hour urine collection, processed in centralized laboratories with commercial kits and sophisticated instruments, a method that can take several days to yield results [25]. This approach is often inconvenient, costly, and labor-intensive, highlighting the need for more efficient POC testing methods.

Recent advancements in POC devices for urinary protein detection include microfluidic biosensors, 3D printing technologies, and smartphone integration. However, many existing techniques need help to combine sensitive, low-cost, high throughput, and rapid detection in a portable format, typically requiring benchtop instruments for signal readout (**Table S5**). In contrast to existing methodologies, our approach attains a notably reduced detection threshold through the optimization of various parameters, including calibrated smartphone exposure times (1/100 s), sample volumes (5 µL), and color channels (red channel) to enable meticulous quantitative analysis with heightened sensitivity.

The pervasive use of smartphones, with approximately 5.22 billion mobile phone users globally, has rendered them an accessible and versatile tool [26]. The affordability of smartphones, bolstered by advancements in optoelectrical sensors, has facilitated their incorporation into a range of applications [27]. Equipped with high-resolution cameras, smartphones have been employed as portable instruments for tasks such as stripe reading [28], microscopy [29], plate reading [30], and imaging flow cytometry [31]. Additionally, smartphones' computational capabilities allow them to perform conventional computational tasks previously restricted to computers. Numerous smartphone-based sensing and imaging techniques have emerged for various applications [32]. Besides, centrifugal microfluidics have gained prominence due to their pumpless operation, robustness, simplicity, and the ability to pre-store reagents [8,33]. Consequently, integrating a homemade spinning device with centrifugal microfluidics and smartphones offers a pump-free system ideal for POC biomarker testing in remote and resource-limited environments. This approach provides a cost-effective, portable, and electrically independent solution for on-site testing.

Two additional models (iPhone 13 Pro and Xiaomi Mi 10T Pro) were analyzed to evaluate the effect of different smartphone models on quantitative detection. The relatively low CV (<3%) of transmittance intensity in the red, green, and blue channels demonstrated the adaptability of different smartphone models for optical imaging and color sensing (**Figure S13**). Additionally, the successfully established calibration curves with high R^2 values (0.9989 and 0.9982) served as internal references to diminish variations between different smartphone models, ensuring reliable quantitative results (**Figure S14**). Furthermore, we compared spinning outcomes across three operators to evaluate user performance consistency. The findings revealed no significant discrepancies, demonstrating the system's robustness (**Figures S15 and S16**).

Urine is a rich source of biomarkers, including proteins, cells, and genes. Still, detecting cell-based and gene-based biomarkers, such as exfoliated bladder cancer cells, presents significant challenges. In contrast, urinary protein biomarkers have been effectively utilized to detect cardiovascular diseases and various cancers [34,35]. Current research efforts are directed toward advancing magnetic bead-assisted microfluidic immunoassay technologies for identifying specific protein biomarkers, such as C-reactive protein. Additionally, urine metabolomic studies have emerged as potent tools for disease detection [36]. Integrating urinary creatinine, a key metabolite, into analytical systems promises to enhance colorimetric assays based on metal nanoparticles' localized surface plasmon resonance, thereby providing a more comprehensive assessment of proteinuria.

Acronyms

UPCR: urine protein to creatinine ratio, UACR: urine albumin to creatinine ratio, POC: point-of-care, ASSURED: Affordable, Sensitive, Specific, User-friendly, Robust and rapid, Equipment-free, Deliverable, HSP-C^M: handheld spinning platforms with centrifugal microfluidics, MI: myocardial infarction, PDMS: polydimethylsiloxanes, CV: coefficient of variation, ET: exposure time, 4-PL: 4-parameter logistic, ROC: receiver operating characteristic, AUC: area under the curve, PMMA: polymethylmethacrylate

Supplementary Figures

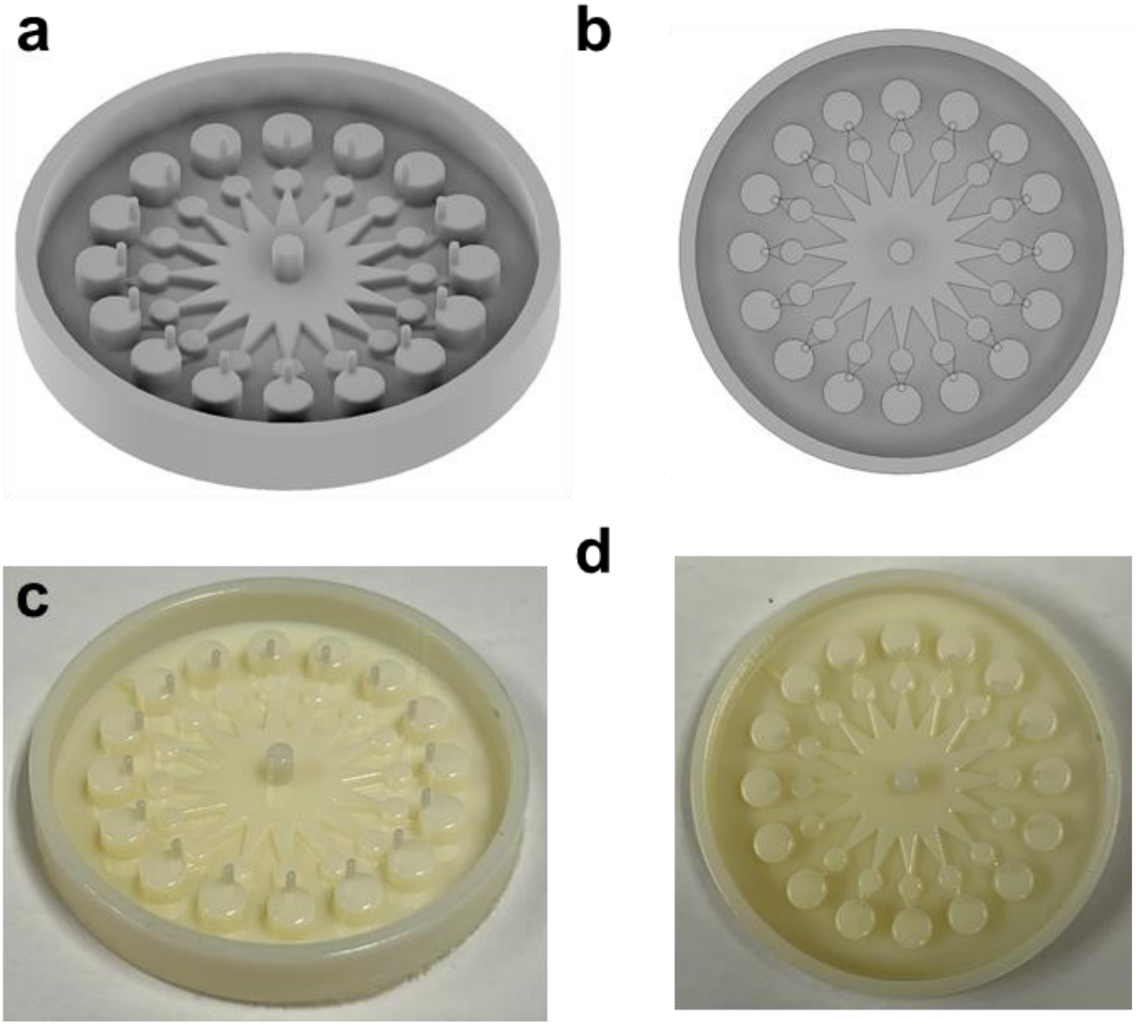

Figure S1. Centrifugal microfluidics mold. (a) 3D schematic diagram of the centrifugal microfluidics mold. (b) Schematics of the mold from a top view. (c) The photo of the mold. (d) The photo of the mold from a top view.

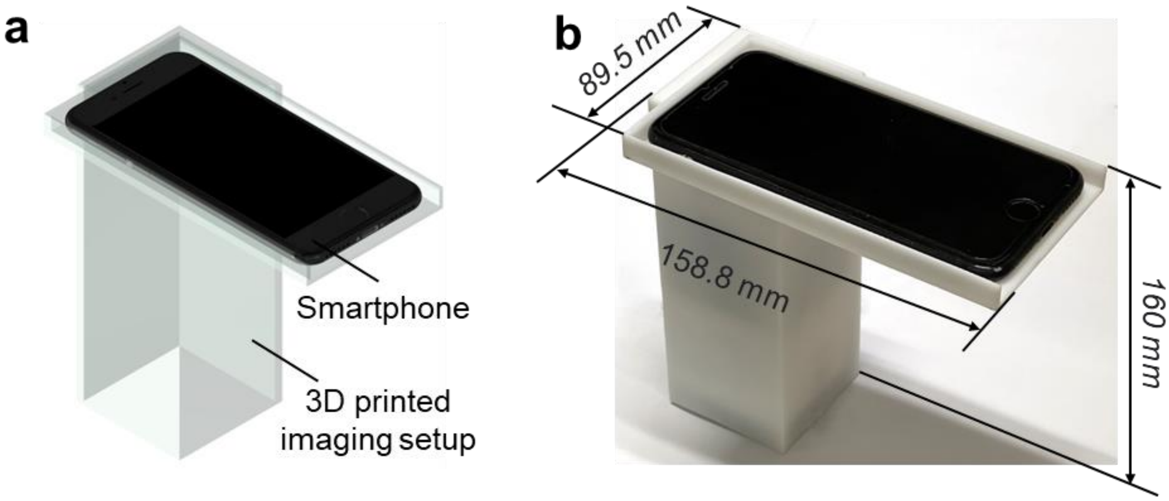

Figure S2. Design of the smartphone-based imaging setup. (a) Schematic diagram of the imaging setup. (b) Photograph of the imaging setup with 158.8 × 89.5 × 160 mm dimensions.

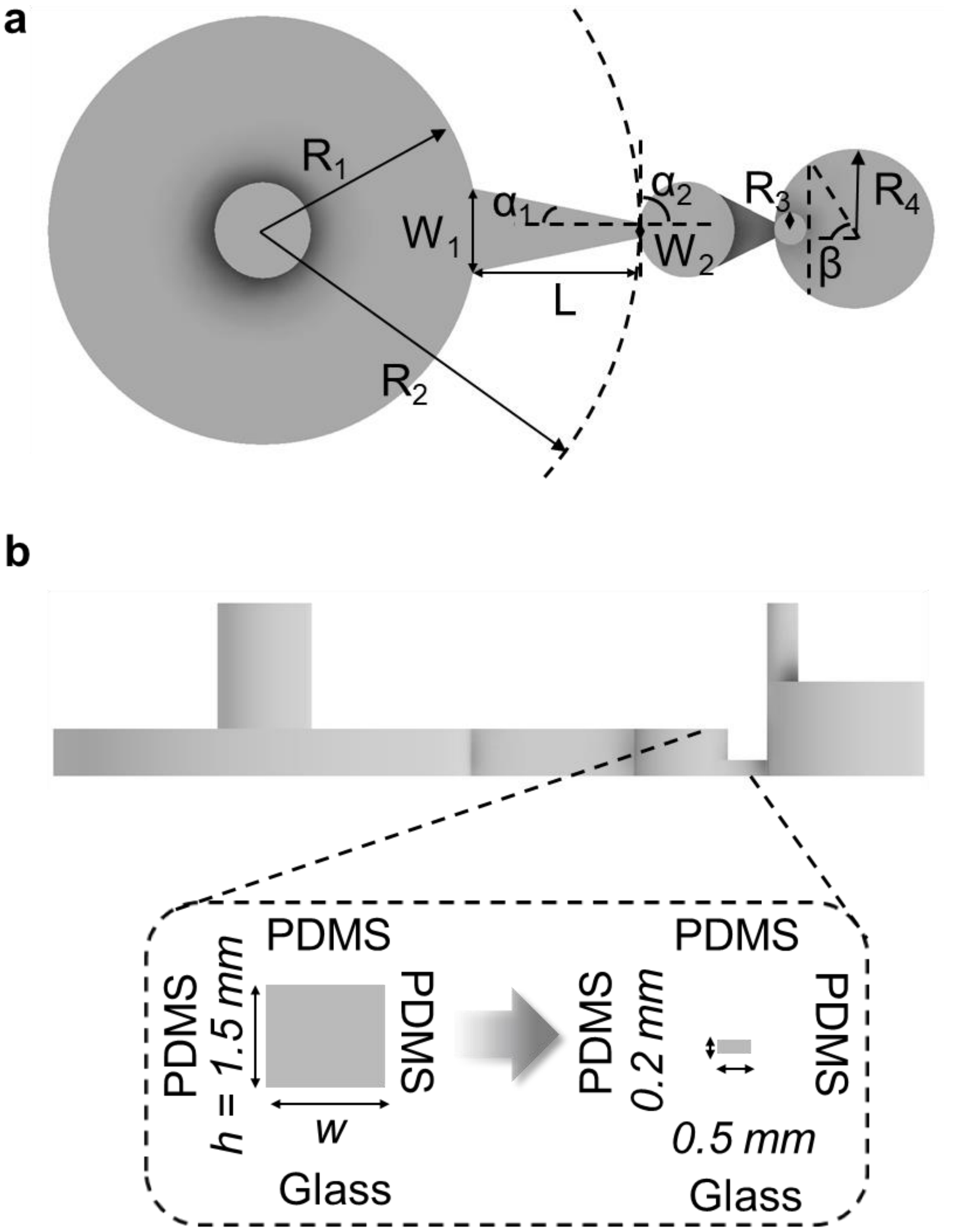

Figure S3. Illustration of centrifugal microfluidics from (a) top view and (b) side view.

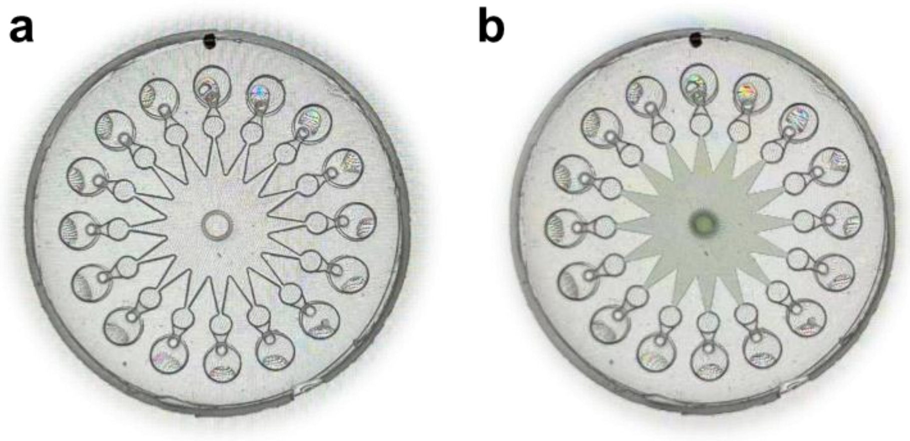

Figure S4. (a) Sample and (b) reagent loading into the HSP-C^M to test samples.

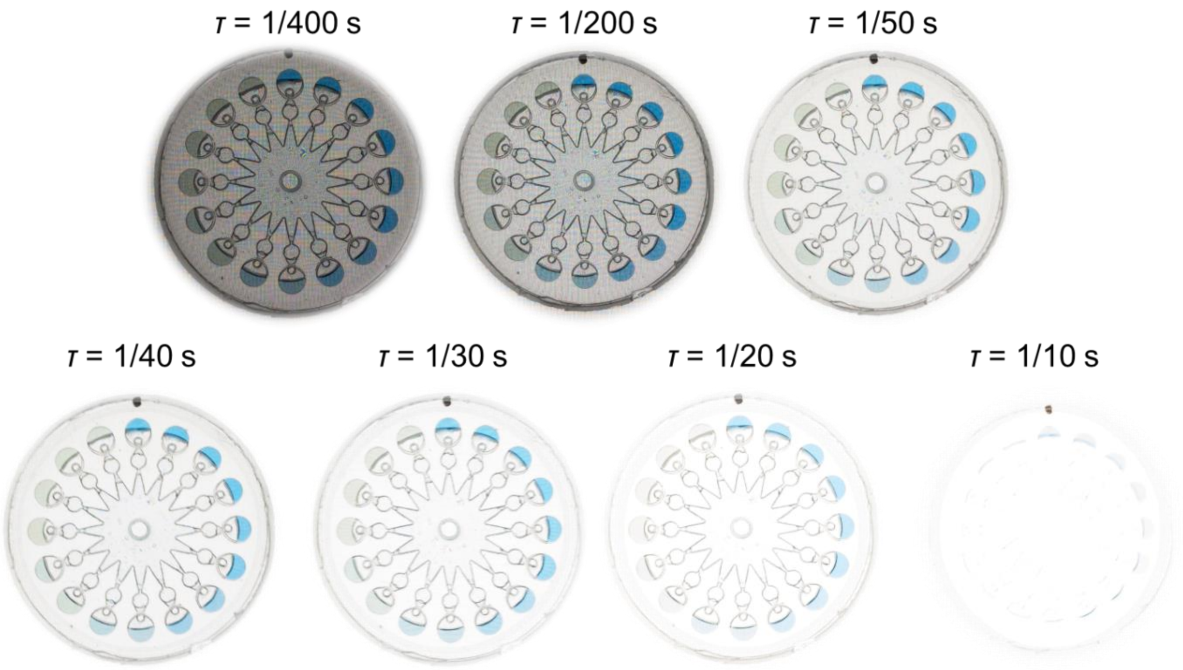

Figure S5. Representative photos of the centrifugal biochip of standards imaged under different exposure times (i.e., 1/400, 1/200, 1/50, 1/40, 1/30, 1/20, and 1/10 s).

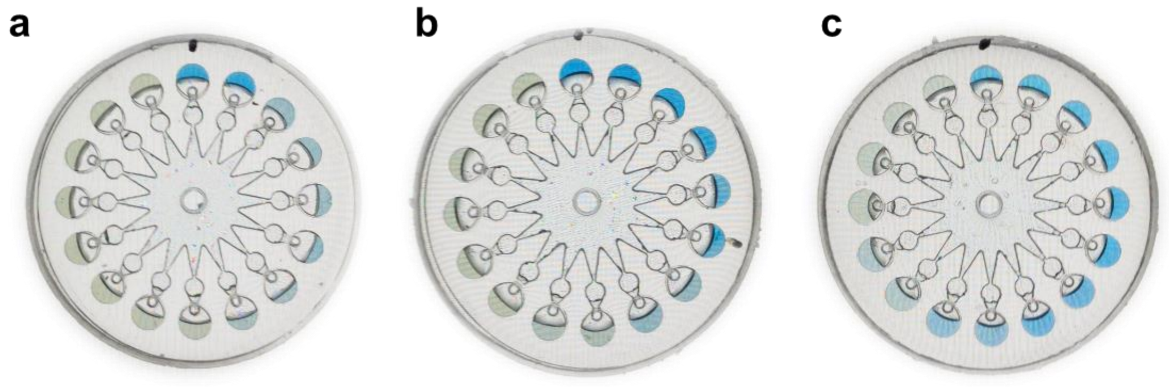

Figure S6. Representative photos of the centrifugal biochip of standards with a sample volume of (a) 1.25 µL, (b) 2.5 µL, and (c) 10 µL, imaged under the exposure time of 1/100 s.

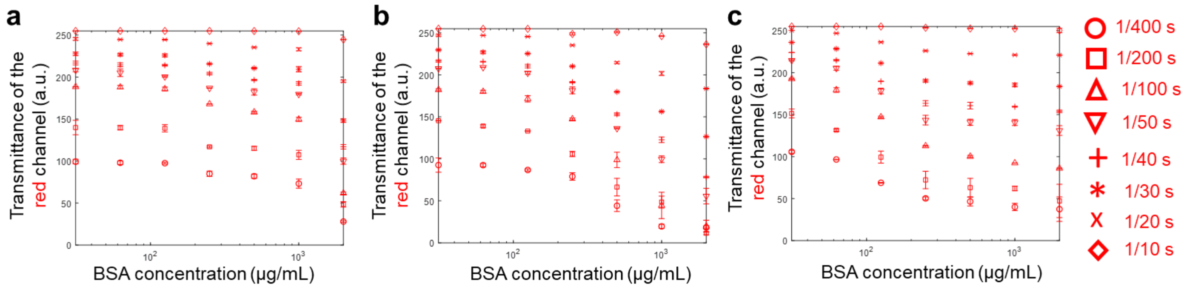

Figure S7. The transmittance of the red channel with the BSA concentration increased. Photos were imaged under different exposure times (i.e., 1/400, 1/200, 1/100, 1/50, 1/40, 1/30, 1/20, and 1/10 s). The sample volumes of BSA are (a) 1.25 µL, (b) 2.5 µL, and (c) 10 µL, respectively.

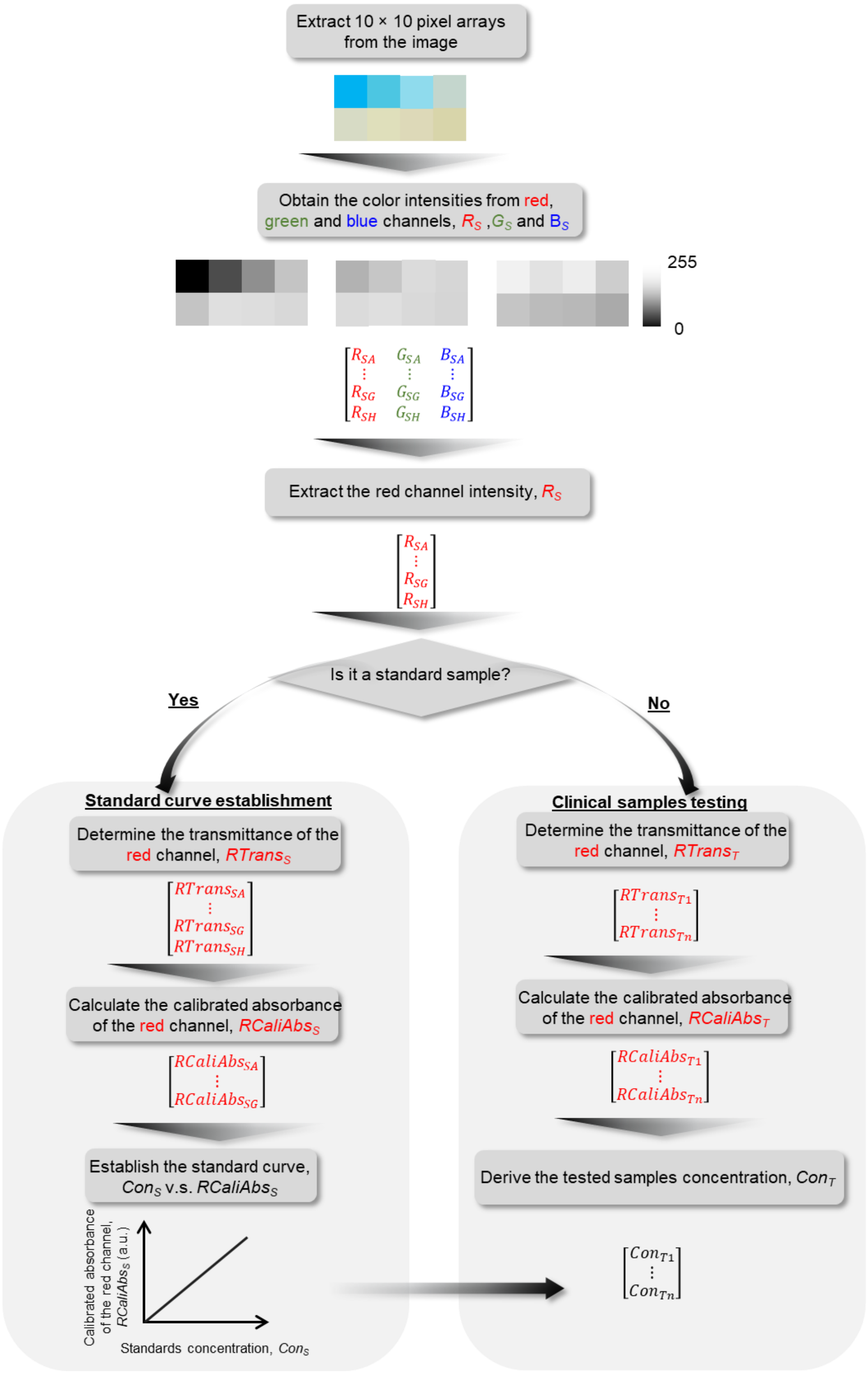

Figure S8. Flowchart of the colorimetric algorithmic analysis procedures.

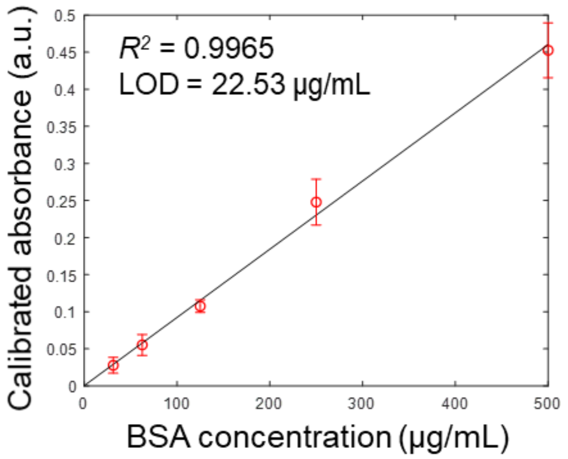

Figure S9. The calibration curve of standards was detected by the plate reader with a detection limit of 22.53 μg/mL and linear dynamic ranges of 75.90 – 500 μg/mL.

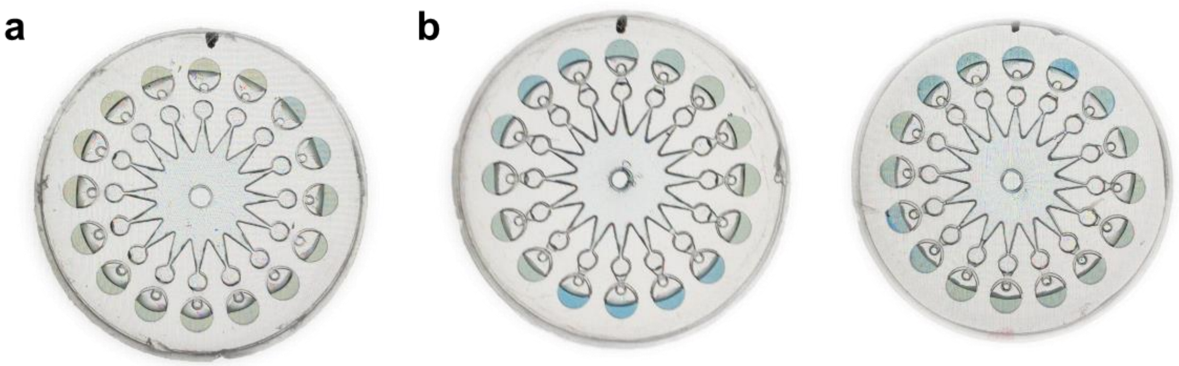

Figure S10. Representative photos of the colorimetric results detected from (a) the healthy cohort and (b) the patient cohort.

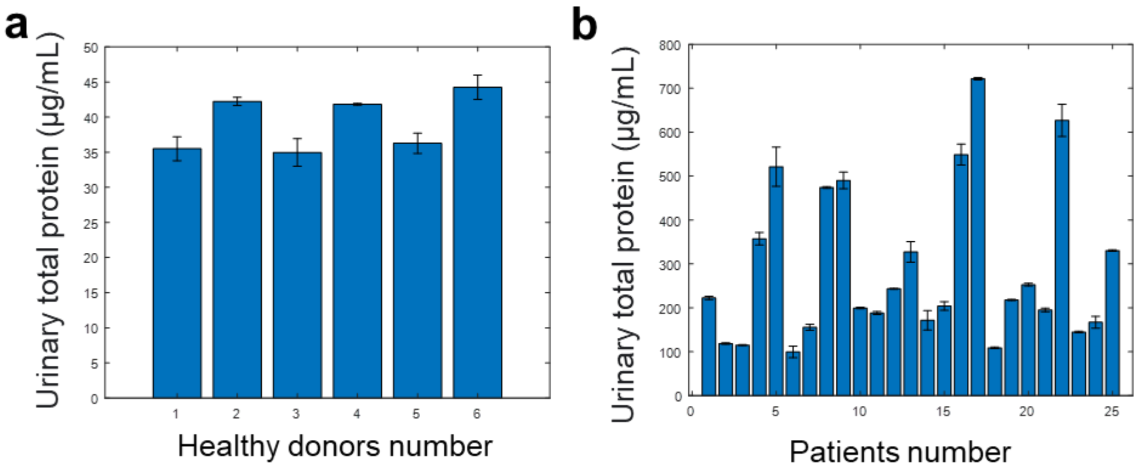

Figure S11. Clinical testing results of the UTP levels from (a) healthy donors (n = 6) and (b) patients after myocardial infarction (MI) surgery (n = 25) detected by commercial plate reader.

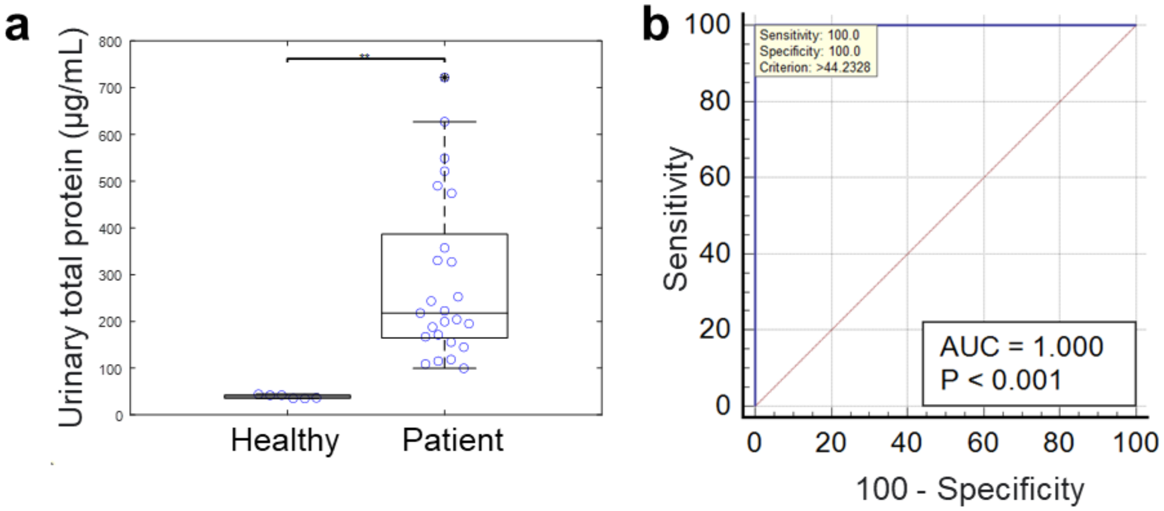

Figure S12. Correlation analysis of clinical urine samples detected by the commercial plate reader. (a) The boxplot of UTP levels from healthy donors (n = 6, 39.16 ± 3.69 μg/mL) and patients after MI surgery (n = 25, 288.08 ± 172.95 μg/mL). (b) Corresponding received operator curves of (a). The optimal sensitivity, specificity, cutoff values, and AUC were shown in the plots.

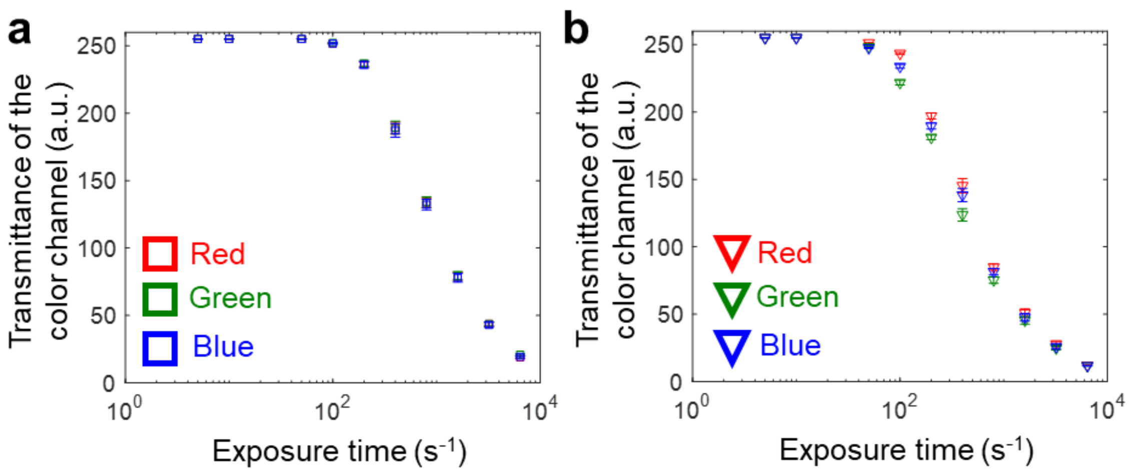

Figure S13. The transmittance intensity of the illumination detected by (a) iPhone 13 Pro and (b) Xiaomi Mi 10T Pro. The color intensities decreased as the smartphone exposure time was reduced.

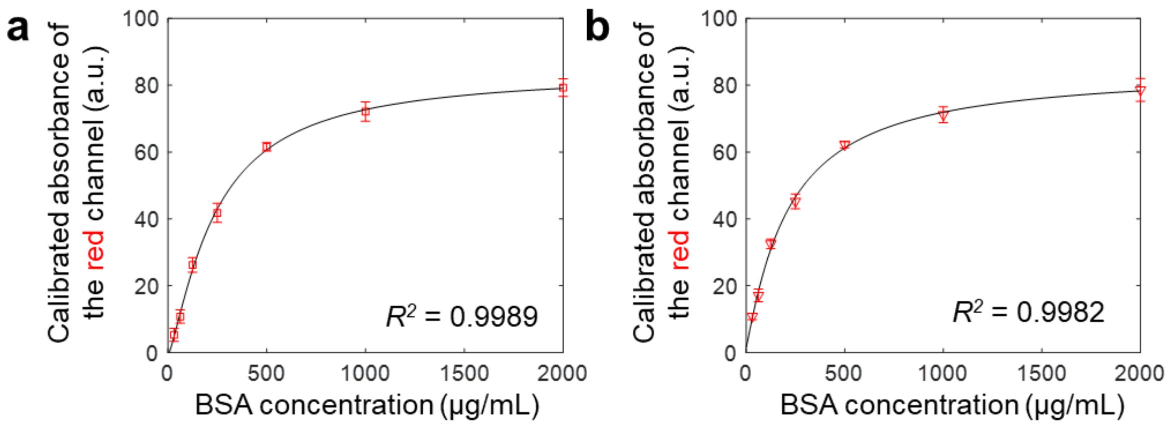

Figure S14. Established calibration curves of standard samples from (a) iPhone 13 Pro and (b) Xiaomi Mi 10T Pro with exposure times of 1/100 s. The calibration curve formulas are $y = \frac{84.93}{1+\left(\frac{x}{237.94}\right)^{-1.30}} - 0.84$, and $y = \frac{83.30}{1+\left(\frac{x}{216.44}\right)^{-1.11}} + 1.56$, respectively. The R^2 values are shown in the figures.

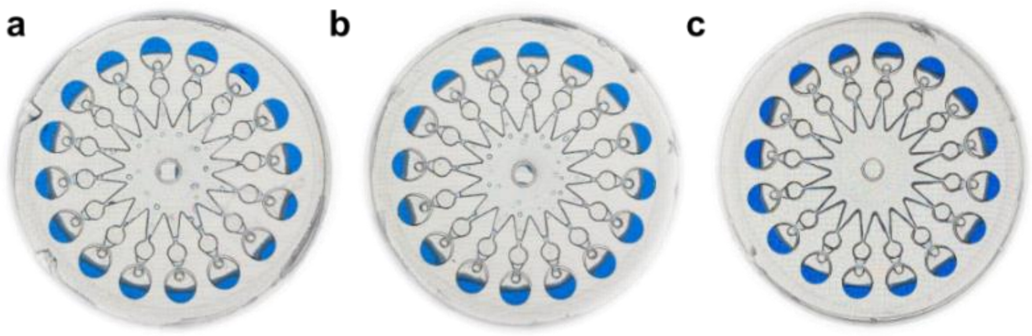

Figure S15. Representative photographs of solution distribution tested by three different operators (a)–(c).

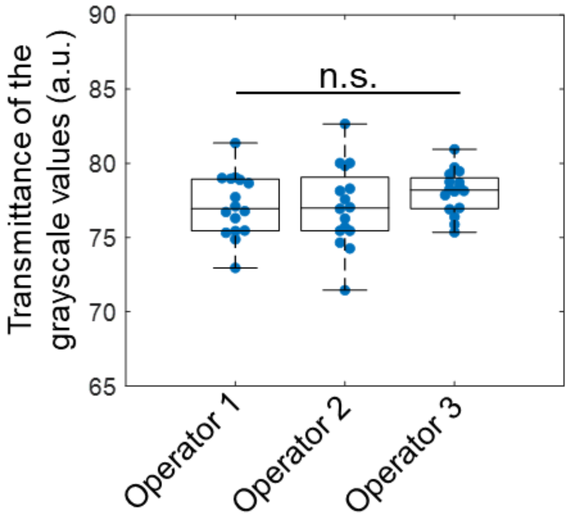

Figure S16. Detected transmittance results were based on grayscale values (CV = 2.64 ± 0.65%) from three operators (n = 3).

Supplementary Tables

Table S1. Comparison of advantages and limitations of conventional techniques for proteinuria measurement.

Technique	Qualitative	Instrument	Detection limit	Sample volume	Time	Cost	Refs
Urinary dipstick	No	Sophisticated	High	~ mL	Quick	Low	[37,38]
UPCR/UACR calculation	Yes	Sophisticated	Medium	~ mL	Medium	Medium	[39,40]
24-hour urine tests	Yes	Sophisticated	Medium	~ mL	24 hours	Medium	[41]
POC urinary protein/albumin test	Yes	Medium/Portable	Low	Low (~ μL)	Quick	Low	This work

Table S2. The disposable centrifugal microfluidic chip cost.

Component	Unit cost (US$)	Quantity	Total cost (US$)
Top fluidic delivery layer	0.98	1	0.98
Bottom substrate layer	0.42	1	0.42
		Total	1.4

Table S3. Assay cost per chip.

Reagent	Price (US$)	Total amount	Amount/ chip	Cost/ chip (US$)
5x G250 dye	28.06	100 mL	410 µL (1x G250 dye)	0.02
			Total	0.02

Table S4. Comparison of existing centrifugal microfluidic techniques with our system. ND = not determined.

Technique	Driving forces	Throughput	Cost	Microfluidic materials	Fabrication difficulty	Detected analyte	Refs
Centrifugal disk-based micro-total analysis system	Electricity	n = 8	ND	PMMA and adhesive layers (5 layers)	Relatively high	Bacterial pathogen	42
Lab-on-compact-disc	Electricity	n = 4	ND	PMMA and adhesive layers (3 layers)	Middle	Antibody	43
Handyfuge microfluidic	Electricity	n = 12	ND	PMMA and adhesive layers (4 layers)	Middle	Pathogenic bacteria	4
Hand-powered centrifugal microfluidic platform	Spinning top	n = 8	ND	PMMA and adhesive layers (4 layers)	Middle	Pathogenic bacteria	2
HSP-C^{M}	Hanheld spinning devices	n = 16	~ 1.5 USD	PDMS and glass layers (2 layers)	Easy	Urinary total protein	Our work

Table S5. Comparison of existing techniques for portable and quantitative urinary protein/albumin test. ND = not determined.

Technique	Disease	Biomarker	Driving forces	Detection limit	Sample volume	Detection time	Cost	Throughput	Clinical samples	Refs
Miniaturized optoelectronic biosensor	Diabetes	Urinary total protein	ND	23 μg/mL	150 μL	< 1s	69-107 USD	n = 1	n = 14	23
3D-printed microfluidic components	ND	Urinary total protein	Torque-actuated pump	8.5 μg/mL	15 μL	25 min	0.22 USD	n = 1	ND	44
Passive mixing microfluidic urinary albumin chip	Chronic kidney diseases	Urinary albumin	Gravitational forces	5.2 μg/mL	10 μL	<5 min	<1 USD	n = 16	n = 12	45
HSP-C^M	After myocardial infarction surgery	Urinary total protein	Centrifugal forces	3.49 μg/mL	5 μL	~ 3 min	~ 1.4 USD	n = 16	n = 31	Our work

References

(1) Bhamla, M. S.; Benson, B.; Chai, C.; Katsikis, G.; Johri, A.; Prakash, M. Hand-Powered Ultralow-Cost Paper Centrifuge. *Nat. Biomed. Eng.* **2017**, *1*, 0009.

(2) Zhang, L.; Tian, F.; Liu, C.; Feng, Q.; Ma, T.; Zhao, Z.; Li, T.; Jiang, X.; Sun, J. Hand-Powered Centrifugal Microfluidic Platform Inspired by the Spinning Top for Sample-to-Answer Diagnostics of Nucleic Acids. *Lab Chip* **2018**, *18*, 610-619.

(3) Gong, F.; Wei, H.-x.; Qi, J.; Ma, H.; Liu, L.; Weng, J.; Zheng, X.; Li, Q.; Zhao, D.; Fang, H. Pulling-Force Spinning Top for Serum Separation Combined with Paper-Based Microfluidic Devices in Covid-19 Elisa Diagnosis. *ACS Sens.* **2021**, *6*, 2709-2719.

(4) Li, S.; Wan, C.; Wang, B.; Chen, D.; Zeng, W.; Hong, X.; Li, L.; Pang, Z.; Du, W.; Feng, X. Handyfuge Microfluidic for on-Site Antibiotic Susceptibility Testing. *Anal. Chem.* **2023**, *95*, 6145-6155.

(5) Lin, C.-T.; Kuo, S.-H.; Lin, P.-H.; Chiang, P.-H.; Lin, W.-H.; Chang, C.-H.; Tsou, P.-H.; Li, B.-R. Hand-Powered Centrifugal Microfluidic Disc with Magnetic Chitosan Bead-Based Elisa for Antibody Quantitation. *Sensor. Actuat. B-Chem.* **2020**, *316*, 128003.

(6) Michael, I.; Kim, D.; Gulenko, O.; Kumar, S.; Kumar, S.; Clara, J.; Ki, D. Y.; Park, J.; Jeong, H. Y.; Kim, T. S. A Fidget Spinner for the Point-of-Care Diagnosis of Urinary Tract Infection. *Nat. Biomed. Eng.* **2020**, *4*, 591-600.

(7) Michael, I. J.; Kim, T.-H.; Sunkara, V.; Cho, Y.-K. Challenges and Opportunities of Centrifugal Microfluidics for Extreme Point-of-Care Testing. *Micromachines-Basel* **2016**, *7*, 32.

(8) Gorkin, R.; Park, J.; Siegrist, J.; Amasia, M.; Lee, B. S.; Park, J.-M.; Kim, J.; Kim, H.; Madou, M.; Cho, Y.-K. Centrifugal Microfluidics for Biomedical Applications. *Lab Chip* **2010**, *10*, 1758-1773.

(9) Kim, T.-H.; Lim, M.; Park, J.; Oh, J. M.; Kim, H.; Jeong, H.; Lee, S. J.; Park, H. C.; Jung, S.; Kim, B. C. Fast: Size-Selective, Clog-Free Isolation of Rare Cancer Cells from Whole Blood at a Liquid–Liquid Interface. *Anal. Chem.* **2017**, *89*, 1155-1162.

(10) Kim, C.-J.; Park, J.; Sunkara, V.; Kim, T.-H.; Lee, Y.; Lee, K.; Kim, M.-H.; Cho, Y.-K. Fully Automated, on-Site Isolation of Cfdna from Whole Blood for Cancer Therapy Monitoring. *Lab Chip* **2018**, *18*, 1320-1329.

(11) Woo, H.-K.; Sunkara, V.; Park, J.; Kim, T.-H.; Han, J.-R.; Kim, C.-J.; Choi, H.-I.; Kim, Y.-K.; Cho, Y.-K. Exodisc for Rapid, Size-Selective, and Efficient Isolation and Analysis of Nanoscale Extracellular Vesicles from Biological Samples. *ACS Nano* **2017**, *11*, 1360-1370.

(12) Miyazaki, C. M.; Carthy, E.; Kinahan, D. J. Biosensing on the Centrifugal Microfluidic Lab-on-a-Disc Platform. *Processes* **2020**, *8*, 1360.

(13) Bhattacharjee, N.; Urrios, A.; Kang, S.; Folch, A. The Upcoming 3d-Printing Revolution in Microfluidics. *Lab Chip* **2016**, *16*, 1720-1742.

(14) Dai, B.; Zhang, L.; Zhao, C.; Bachman, H.; Becker, R.; Mai, J.; Jiao, Z.; Li, W.; Zheng, L.; Wan, X. Biomimetic Apposition Compound Eye Fabricated Using Microfluidic-Assisted 3d Printing. *Nat. Commun.* **2021**, *12*, 6458.

(15) Liao, J.; Ma, Z.; Liu, S.; Li, W.; Yang, X.; Hilal, M. E.; Zhou, X.; Yang, Z.; Khoo, B. L. Programmable Microfluidic-Assisted Highly Conductive Hydrogel Patches for Customizable Soft Electronics. *Adv. Funct. Mater.* **2024**, *34*, 2401930.

(16) Deckert, T.; Feldt-Rasmussen, B.; Borch-Johnsen, K.; Jensen, T.; Kofoed-Enevoldsen, A. Albuminuria Reflects Widespread Vascular Damage: The Steno Hypothesis. *Diabetologia* **1989**, *32*, 219-226.

(17) Currie, G.; Delles, C. Proteinuria and Its Relation to Cardiovascular Disease. *Int. J. Nephrol. Renovasc. Dis.* **2013**, 13-24.

(18) Tsioufis, C.; Dimitriadis, K.; Chatzis, D.; Vasiliadou, C.; Tousoulis, D.; Papademetriou, V.; Toutouzas, P.; Stefanadis, C.; Kallikazaros, I. Relation of Microalbuminuria to Adiponectin and Augmented C-Reactive Protein Levels in Men with Essential Hypertension. *Am. J. Cardiol.* **2005**, *96*, 946-951.

(19) Zhu, X.; Wu, S.; Dahut, W. L.; Parikh, C. R. Risks of Proteinuria and Hypertension with Bevacizumab, an Antibody against Vascular Endothelial Growth Factor: Systematic Review and Meta-Analysis. *Am. J. Kidney Dis.* **2007**, *49*, 186-193.
(20) Mykkänen, L.; Zaccaro, D. J.; Wagenknecht, L. E.; Robbins, D. C.; Gabriel, M.; Haffner, S. M. Microalbuminuria Is Associated with Insulin Resistance in Nondiabetic Subjects: The Insulin Resistance Atherosclerosis Study. *Diabetes* **1998**, *47*, 793-800.
(21) Kanbayashi, Y.; Ishikawa, T.; Tabuchi, Y.; Sakaguchi, K.; Ouchi, Y.; Otsuji, E.; Takayama, K.; Taguchi, T. Predictive Factors for the Development of Proteinuria in Cancer Patients Treated with Bevacizumab, Ramucirumab, and Aflibercept: A Single-Institution Retrospective Analysis. *Sci. Rep.* **2020**, *10*, 2011.
(22) Lamb, E. J.; MacKenzie, F.; Stevens, P. E. How Should Proteinuria Be Detected and Measured? *Ann. Clin. Biochem.* **2009**, *46*, 205-217.
(23) Hashemipour, S.; Charkhchian, M.; Javadi, A.; Afaghi, A.; Hajiaghamohamadi, A. A.; Bastani, A.; Hajmanoochehri, F.; Ziaee, A. Urinary Total Protein as the Predictor of Albuminuria in Diabetic Patients. *Int. J. Endocrinol Met.* **2012**, *10*, 523.
(24) Mahzabeen, F.; Vermesh, O.; Levi, J.; Tan, M.; Alam, I. S.; Chan, C. T.; Gambhir, S. S.; Harris, J. S. Real-Time Point-of-Care Total Protein Measurement with a Miniaturized Optoelectronic Biosensor and Fast Fluorescence-Based Assay. *Biosens. Bioelectron.* **2021**, *180*, 112823.
(25) Li, H.; Shkolyar, E.; Wang, J.; Conti, S.; Pao, A. C.; Liao, J. C.; Wong, T.-S.; Wong, P. K. Slips-Lab—a Bioinspired Bioanalysis System for Metabolic Evaluation of Urinary Stone Disease. *Sci. Adv.* **2020**, *6*, eaba8535.
(26) Kemp, S. Digital 2021: Global Overview Report. Datareportal. 2021.
(27) Ozcan, A. Mobile Phones Democratize and Cultivate Next-Generation Imaging, Diagnostics and Measurement Tools. *Lab Chip* **2014**, *14*, 3187-3194.
(28) Smith, G. T.; Dwork, N.; Khan, S. A.; Millet, M.; Magar, K.; Javanmard, M.; Bowden, A. K. E. Robust Dipstick Urinalysis Using a Low-Cost, Micro-Volume Slipping Manifold and Mobile Phone Platform. *Lab Chip* **2016**, *16*, 2069-2078.
(29) Zhang, J.; Li, W.; Huang, Q.; Fu, Y.; Liu, Y.; Luo, X.; Zou, S.; Chua, S. L.; Leung, S.; Khoo, B. L. Label-Free Biosensor for Non-Invasive and Low-Cost Detection of Metastatic Risk through Urine Biopsy. *Sensor. Actuat. B-Chem.* **2023**, *395*, 134485.
(30) Berg, B.; Cortazar, B.; Tseng, D.; Ozkan, H.; Feng, S.; Wei, Q.; Chan, R. Y.-L.; Burbano, J.; Farooqui, Q.; Lewinski, M. Cellphone-Based Hand-Held Microplate Reader for Point-of-Care Testing of Enzyme-Linked Immunosorbent Assays. *ACS Nano* **2015**, *9*, 7857-7866.
(31) Aslan, M. K.; Ding, Y.; Stavrakis, S.; deMello, A. J. Smartphone Imaging Flow Cytometry for High-Throughput Single-Cell Analysis. *Anal. Chem.* **2023**, *95*, 14526-14532.
(32) Zhang, D.; Liu, Q. Biosensors and Bioelectronics on Smartphone for Portable Biochemical Detection. *Biosens. Bioelectron.* **2016**, *75*, 273-284.
(33) Strohmeier, O.; Keller, M.; Schwemmer, F.; Zehnle, S.; Mark, D.; von Stetten, F.; Zengerle, R.; Paust, N. Centrifugal Microfluidic Platforms: Advanced Unit Operations and Applications. *Chem. Soc. Rev.* **2015**, *44*, 6187-6229.
(34) Röthlisberger, S.; Pedroza-Diaz, J. Urine Protein Biomarkers for Detection of Cardiovascular Disease and Their Use for the Clinic. *Expert Rev. Proteomic.* **2017**, *14*, 1091-1103.
(35) Casado-Vela, J.; Del Pulgar, T. G.; Cebrian, A.; Alvarez-Ayerza, N.; Lacal, J. C. Human Urine Proteomics: Building a List of Human Urine Cancer Biomarkers. *Expert Rev. Proteomic.* **2011**, *8*, 347-360.
(36) Dinges, S. S.; Hohm, A.; Vandergrift, L. A.; Nowak, J.; Habbel, P.; Kaltashov, I. A.; Cheng, L. L. Cancer Metabolomic Markers in Urine: Evidence, Techniques and Recommendations. *Nat. Rev. Urol.* **2019**, *16*, 339-362.
(37) Meyer, N. L.; Mercer, B. M.; Friedman, S. A.; Sibai, B. M. Urinary Dipstick Protein: A Poor Predictor of Absent or Severe Proteinuria. *Am. J. Obstet. Gynecol.* **1994**, *170*, 137-141.
(38) Simerville, J. A.; Maxted, W. C.; Pahira, J. J. Urinalysis: A Comprehensive Review. *Am. Fam. Physician* **2005**, *71*, 1153-1162.

(39) Fotheringham, J.; Campbell, M. J.; Fogarty, D. G.; El Nahas, M.; Ellam, T. Estimated Albumin Excretion Rate Versus Urine Albumin-Creatinine Ratio for the Estimation of Measured Albumin Excretion Rate: Derivation and Validation of an Estimated Albumin Excretion Rate Equation. *Am. J. Kidney Dis.* **2014**, *63*, 405-414.
(40) Jia, Y.; Liu, G.; Xu, G.; Li, X.; Shi, Z.; Cheng, C.; Xu, D.; Lu, Y.; Liu, Q. Battery-Free and Wireless Tag for in Situ Sensing of Urinary Albumin/Creatinine Ratio (Acr) for the Assessment of Albuminuria. *Sensor. Actuat. B-Chem.* **2022**, *367*, 132050.
(41) Côté, A.-M.; Firoz, T.; Mattman, A.; Lam, E. M.; von Dadelszen, P.; Magee, L. A. The 24-Hour Urine Collection: Gold Standard or Historical Practice? *Am. J. Obstet. Gynecol.* **2008**, *199*, 625. e621-625. e626.
(42) Noroozi, Z.; Kido, H.; Peytavi, R.; Nakajima-Sasaki, R.; Jasinskas, A.; Micic, M.; Felgner, P. L.; Madou, M. J. A Multiplexed Immunoassay System Based Upon Reciprocating Centrifugal Microfluidics. *Rev. Sci. Instrum.* **2011**, *82*.
(43) Thiha, A.; Ibrahim, F. A Colorimetric Enzyme-Linked Immunosorbent Assay (Elisa) Detection Platform for a Point-of-Care Dengue Detection System on a Lab-on-Compact-Disc. *Sensors-Basel* **2015**, *15*, 11431-11441.
(44) Chan, H. N.; Shu, Y.; Xiong, B.; Chen, Y.; Chen, Y.; Tian, Q.; Michael, S. A.; Shen, B.; Wu, H. Simple, Cost-Effective 3d Printed Microfluidic Components for Disposable, Point-of-Care Colorimetric Analysis. *ACS Sens.* **2016**, *1*, 227-234.
(45) Wu, J.; Tomsa, D.; Zhang, M.; Komenda, P.; Tangri, N.; Rigatto, C.; Lin, F. A Passive Mixing Microfluidic Urinary Albumin Chip for Chronic Kidney Disease Assessment. *ACS Sens.* **2018**, *3*, 2191-2197.
